# Extracellular Tuning of Mitochondrial Respiration Leads to Aortic Aneurysm

**DOI:** 10.1161/CIRCULATIONAHA.120.051171

**Published:** 2021-03-12

**Authors:** Jorge Oller, Enrique Gabandé-Rodríguez, María Jesús Ruiz-Rodríguez, Gabriela Desdín-Micó, Juan Francisco Aranda, Raquel Rodrigues-Diez, Constanza Ballesteros-Martínez, Eva María Blanco, Raquel Roldan-Montero, Pedro Acuña, Alberto Forteza Gil, Carlos E. Martín-López, J. Francisco Nistal, Christian L. Lino Cardenas, Mark Evan Lindsay, José Luís Martín-Ventura, Ana M. Briones, Juan Miguel Redondo, María Mittelbrunn

**Affiliations:** 1 Departamento de Biología Molecular, Centro de Biología Molecular Severo Ochoa, Consejo Superior de Investigaciones Científicas Universidad Autónoma de Madrid, Spain (J.O., E.G-R., G.D-M., J.F.A., E.M.B., P.A., M.M.).; 2 Instituto de Investigación Sanitaria del Hospital 12 de Octubre (i+12), Madrid, Spain (J.O., E.G-R., G.D-M., J.F.A., E.M.B., M.M.).; 3 Centro de Investigación Biomédica en Red de Enfermedades Cardiovasculares, Spain (J.O., R.R-D., R.R-M., A.M.B., J.M.R.).; 4 Centro Nacional de Investigaciones Cardiovasculares Carlos III, Madrid, Spain (M.J.R-R., J.M.R.).; 5 Departamento de Farmacología, Universidad Autónoma de Madrid, Instituto de Investigación Hospital La Paz, Spain (R.R-D., C.B-M., A.M.B.).; 6 Instituto de Investigación Sanitaria-Fundación Jimenez Diaz, Madrid, Spain (R.R-M. J.L.M-V.).; 7 Hospital Universitario Puerta de Hierro, Madrid, Spain. (R.R-M., J.L.M-V.).; 8 Cardiovascular Surgery, Hospital Universitario Marqués de Valdecilla, IDIVAL, Universidad de Cantabria, Santander, Spain. (J.F.N.).; 9 Massachusetts General Hospital Thoracic Aortic Center, Boston (C.L.L.C., M.E.L.).

**Keywords:** aortic aneurysm, DNA, mitochondrial, extracellular matrix, genetic diseases, inborn, glycolysis, Marfan syndrome, muscle, smooth, vascular

## Abstract

**Background:**

Marfan syndrome (MFS) is an autosomal dominant disorder of the connective tissue caused by mutations in the *FBN1* (fibrillin-1) gene encoding a large glycoprotein in the extracellular matrix called fibrillin-1. The major complication of this connective disorder is the risk to develop thoracic aortic aneurysm. To date, no effective pharmacologic therapies have been identified for the management of thoracic aortic disease and the only options capable of preventing aneurysm rupture are endovascular repair or open surgery. Here, we have studied the role of mitochondrial dysfunction in the progression of thoracic aortic aneurysm and mitochondrial boosting strategies as a potential treatment to managing aortic aneurysms.

**Methods:**

Combining transcriptomics and metabolic analysis of aortas from an MFS mouse model (*Fbn1*^*c1039g/+*^) and MFS patients, we have identified mitochondrial dysfunction alongside with mtDNA depletion as a new hallmark of aortic aneurysm disease in MFS. To demonstrate the importance of mitochondrial decline in the development of aneurysms, we generated a conditional mouse model with mitochondrial dysfunction specifically in vascular smooth muscle cells (VSMC) by conditional depleting Tfam (mitochondrial transcription factor A; *Myh11-Cre*^*ERT2*^*Tfam*^*flox/flox*^ mice). We used a mouse model of MFS to test for drugs that can revert aortic disease by enhancing Tfam levels and mitochondrial respiration.

**Results:**

The main canonical pathways highlighted in the transcriptomic analysis in aortas from *Fbn1*^*c1039g/+*^ mice were those related to metabolic function, such as mitochondrial dysfunction. Mitochondrial complexes, whose transcription depends on Tfam and mitochondrial DNA content, were reduced in aortas from young *Fbn1*^*c1039g/+*^ mice. In vitro experiments in *Fbn1*-silenced VSMCs presented increased lactate production and decreased oxygen consumption. Similar results were found in MFS patients. VSMCs seeded in matrices produced by Fbn1-deficient VSMCs undergo mitochondrial dysfunction. Conditional Tfam-deficient VSMC mice lose their contractile capacity, showed aortic aneurysms, and died prematurely. Restoring mitochondrial metabolism with the NAD precursor nicotinamide riboside rapidly reverses aortic aneurysm in *Fbn1*^*c1039g/+*^ mice.

**Conclusions:**

Mitochondrial function of VSMCs is controlled by the extracellular matrix and drives the development of aortic aneurysm in Marfan syndrome. Targeting vascular metabolism is a new available therapeutic strategy for managing aortic aneurysms associated with genetic disorders.

## Abstract 2

Supplemental Digital Content is available in the text.

**Clinical Perspective**

**What Is New?**

- Tfam and mitochondrial-DNA levels decline correlate with aortic dilation in *Fbn1*^*c1039g/+*^ mice.
- Aortic samples and cells from patients with Marfan syndrome present mitochondrial defects.
- Mitochondrial respiration is fine-tuned by the extracellular matrix.
- Mitochondrial dysfunction in vascular smooth muscle cells promotes aortic dilation, aneurysms, and premature death.
- Boosting mitochondrial function by a NAD^+^ precursor rapidly reverses transcriptional signature and aortic dilation in *Fbn1*^*c1039g/+*^ mice.

**What Are the Clinical Implications?**

- Boosting mitochondrial function with NAD^+^ precursors is a new therapeutic opportunity to manage aortic aneurysms associated with connective genetic disorders and to prevent aortic dissection.

Different inherited disorders affect the structure of the arterial wall and lead to thoracic aortic aneurysms (TAA).^1,2^ Most of these diseases are associated with mutations in genes related to the extracellular matrix (ECM), or the vascular smooth muscle cell (VSMC)–contractile apparatus. Marfan syndrome (MFS) is one of the most commonly inherited disorders affecting connective tissue. It is caused by mutations in the gene that encodes for the extracellular protein FBN1 (fibrillin-1) and has a reported incidence of 1 in 5000 individuals.^3,4^ MFS patients present extended bones, lens luxation, and decreased life expectancy primarily attributable to TAAs.^5^ Mutations in genes that encode proteins involved in TGFβ–signaling cause Loeys–Dietz syndrome, a disease that phenocopies part of the skeletal features of MFS.^6^ Vascular-type Ehlers–Danlos syndrome is mainly caused by mutations in *COL3A1* gene (collagen 3a1) that produce hyperelasticity in joints and skin, small stature, and aortic dissections.^7^ Moreover, the rare condition *Cutis laxa* syndrome is caused by mutations in elastin fibers genes (*ELN* [elastin], *FBLN4* [fibulin 4], *FBLN5* [fibulin 5]) that produce a loss of elastic properties in tissues such as the skin and arteries.^8,9^ Finally, although most of the genes involved in familial forms of nonsyndromic thoracic aortic aneurysm and dissections remain unknown, some of those identified (accounting for only about 20% of all patients with familial forms of nonsyndromic thoracic aortic aneurysm and dissections ) are involved in the maintenance of the smooth muscle contractile apparatus, such as PRKG1 (cGMP-dependent protein kinase 1), *MYLK* (myosin light chain kinase), *MYH11* (myosin heavy chain11), and *ACTA2* (actin α2, smooth muscle).^10^

The major complication of all these connective inherited disorders is the risk to develop TAAs. TAA is a complex vascular pathology characterized by permanent dilation of the thoracic aorta. TAA progression can lead to catastrophic consequences like rupture or dissection of the arterial wall, causing patient death because of extensive hemorrhage. VSMCs are located in the medial layer of arteries and directly drive the contraction of the vascular wall regulating the size of the vessel lumen. VSMCs can shift reversibly from a quiescent or contractile phenotype to a secretory phenotype.^11–13^ Changes toward this secretory phenotype generate pathologic features such as an elevated proliferation rate and increased ECM accumulation favoring medial degeneration in the aneurysm development.^12–15^ ECM remodeling in the aortic wall results in increased aortic stiffness.^16–18^ Studies have started to reveal the connection between cellular adhesion and cytoskeletal reorganization and the metabolism of the cell,^19–21^ supporting that the composition and the stiffness of the ECM regulate cellular metabolism.^22^

Here, we investigate the implication of VSMC metabolism in the progression and development of aortic aneurysm in MFS. We provide evidence that mitochondrial metabolism is a key regulator of VSMC phenotype during aortic remodeling and is fine-tuned by ECM composition. To demonstrate the importance of mitochondrial metabolism in the development of aneurysms, we generated a conditional mouse model with mitochondrial dysfunction specifically in VSMCs by depleting Tfam (mitochondrial transcription factor A). As Tfam controls the transcription, replication, and stability of mitochondrial DNA (mtDNA),^23^ depleting Tfam has been proved to be an effective approach to induce severe mitochondrial dysfunction in different cells and tissues.^24–27^ Tfam-deficient VSMC mice developed aortic dilation, medial degeneration, aortic aneurysm, and fatal dissections, further supporting that mitochondrial function is a key regulator of the VSMC phenotype during aortic remodeling. Finally, we assessed the therapeutic potential of boosting mitochondrial metabolism in MFS. Our results indicate that mitochondrial function could be an effective target for pharmacologic approaches to managing aortic dilation and preventing aortic dissection associated with genetic disorders.

## Methods

The data, analytic methods, and study materials that support the findings of this study are available from the corresponding author on reasonable request.

### Mouse Strains and Animal Procedures

The Marfan mouse model, which harbors a *Fbn1*^*C1039G/+*^ allele (JAX stock No. 012885), was previously described.^28^ For specific ablation of *Tfam* in smooth muscle, we crossed *Tfam*^*flox/flox*^ mice^23^ with mice carrying the *Myh11-Cre*^*ERT2*^ allele (JAX stock No. 019079) that expresses a tamoxifen-inducible Cre recombinase under the regulatory sequences of smooth muscle *Myh11* promoter, inserted into Y-chromosome.^23,29^ Wild type littermates were used as controls for Marfan mice and *Tfam*^*Wt/Wt*^ *Myh11-Cre*^*ERT2*^ for *Tfam*^*flox/flox*^ *Myh11-Cre*^*ERT2*^, unless otherwise specified. For conditional gene deletion, *Myh11-Cre*^*ERT2*^*Tfam*^*Wt/Wt*^ and *Myh11-Cre*^*ERT2*^*Tfam*^*flox/flox*^ 4- to 5-week-old male mice received daily 1-mg IP injections of tamoxifen (Sigma-Aldrich) on 5 consecutive days. Ang II (angiotensin II) dissolved in saline (Sigma-Aldrich) was infused at 1 μg·kg^−1^·min^−1^ using subcutaneous osmotic minipumps (Model 2004, Alzet Corp.). Nicotinamide riboside ([NR] Novalix) was administered intraperitoneally at 1000 mg/kg in saline every other day. Mice were housed at the pathogen-free animal facility of the Centro Nacional de Investigaciones Cardiovasculares Carlos III and Centro de Biología Molecular Severo Ochoa following the animal care standards of the institution. Animal procedures were approved by the Centro Nacional de Investigaciones Cardiovasculares Carlos III and Centro de Biología Molecular Severo Ochoa−Universidad Autonoma de Madrid Ethics Committee and the Madrid regional authorities (ref. PROEX 283/16) and conformed with European Union Directive 2010/63EU and Recommendation 2007/526/EC regarding the protection of animals used for experimental and other scientific purposes, enforced in Spanish law under Real Decreto 1201/2005.

### Blood Pressure Measurements and In Vivo Imaging

Arterial blood pressure (BP) was measured by the mouse tail-cuff method using the automated BP-2000 Blood Pressure Analysis System (Visitech Systems, Apex, NC). Mice were trained for BP measurements every day for 5 consecutive days. After the training period, BP was measured 1 day before treatment to determine the baseline BP values in each mouse cohort. BP measurements were recorded in mice located in a tail-cuff restrainer over a warmed surface (37°C). Fifteen consecutive systolic and diastolic BP measurements were made, and the last 10 readings per mouse were recorded and averaged. For in vivo ultrasound images, the aortic diameter was monitored in isoflurane-anesthetized mice (2% isoflurane) by high-frequency ultrasound with a VEVO 2100 echography device (VisualSonics, Toronto, Canada) at 30-μ resolution. Maximal internal aortic diameters were measured at diastole using VEVO 2100 software, version 1.5.0.

### Cell Procedures

Isolation and culture of primary mouse VSMCs were described in Esteban et al.^30^ Tissue was digested with a solution of collagenase and elastase (Worthington Biochem) until a single-cell suspension was obtained. All experiments with primary VSMCs were performed during passages 2 to 7. Lentiviral transduction was performed in VSMCs during 5 hours at a multiplicity of infection of 3. The medium was then replaced with fresh DMEM supplemented with 10% FBS and cells were cultured for 5 more days, treated with NR for 5 more days, and then serum-starved for 16 hours. The HEK-293T (CRL-1573) and Jurkat (Clone E6-1, TIB-152) cell lines, required for high-titer lentivirus production and lentivirus titration, respectively, were purchased from American Type Culture Collection. Four apparently healthy male controls (GM00024, GM01717, GM03652, GM23963) and 4 males with aged-mismatched fibroblasts from MFS patients with aortic dissection, 2 with *FBN1* point mutations (GM21499, GM21946), and 2 with haploinsufficient *FBN1* mutations (GM21983, GM21978), were purchased to Coriell Cell Repositories. The experiments were performed during passages 5 to 10. All cells tested negative for *Mycoplasma*.

### Lentivirus Production and Infection

The Cre and green fluorescent protein coding sequence was obtained by polymerase chain reaction (PCR) amplification and cloned into the pHRSIN lentiviral vector.^31^ Lentiviruses expressing short hairpin RNA−targeting murine *Fbn1*, and control short hairpin RNA were purchased from Sigma-Aldrich. Pseudo-typed lentiviruses were produced by transient calcium phosphate transfection of HEK-293T cells and concentrated from culture supernatant by ultracentrifugation (2 hours at 128 000*g*; Ultraclear Tubes; SW28 rotor and Optima L-100 XP Ultracentrifuge; Beckman). Viruses were suspended in cold sterile PBS and titrated by transduction of Jurkat cells for 48 hours. Transduction efficiency (green fluorescent protein−expressing cells and puromycin-resistant cells) and cell death (propidium iodide staining) were quantified by flow cytometry.^32^

### Extracellular Flux Analysis and Extracellular L-Lactate Determination

Oxygen consumption rates (OCR) were measured in XF-96 Extracellular Flux Analyzers (Seahorse Bioscience) in 25 000 mouse aortic VSMCs or 50 000 human fibroblasts. Cells were seeded in nonbuffered DMEM medium containing either 25 mM glucose or 1 mM CaCl_2_. Three measurements were obtained under basal conditions and on addition of oligomycin (1 mM), fluoro carbonyl cyanide phenylhydrazone (1.5 mM), and rotenone (100 nmol/L) + antimycin A (1 mM). OCR measurements were normalized to protein cell extracts. Extracellular lactate determination was performed with single-use reagent strips for Accutrend Plus Lactate Pro (Roche), based on an enzymatic spectrophotometry system by lactate oxidase layer. We analyzed 20 µL of conditioned medium after 24 hours of culture. L-lactate was normalized to total protein cell extracts.

### β-Galactosidase Activity Assay

For the β-galactosidase quantitative assay, tissues were lysed with T-PER Tissue Protein Extraction Reagent (78510; Thermo Scientific). Lysates were centrifuged at 10 000*g* for 5 minutes and the supernatant was collected. Fifty microliters of protein lysates were mixed with 50 µL of Pierce β-galactosidase Assay Reagent (75705; Thermo Scientific) for the assay. The reaction was incubated for 1 hour and the absorbance was measured at 415 nm. Values were normalized to total protein extracts.

### ECM Assays

Experiments of ECM were based on Castelló-Cros et al.^33^ First, culture plates were coated with 1% of gelatin (Sigma-Aldrich) and 0.01% collagen I (Gibco) for 1 hour at 37°C. The gelatin was cross-linked by adding 1% glutaraldehyde in PBS and incubated for 30 minutes at 37°C. Then, the plates were washed 3 times for 5 minutes with PBS and 1 additional wash with DMEM. Then, VSMCs were seeded at a confluence of 50% and cultured for 5 days to allow production of the ECM. For the decellularization step, ice-cold extraction buffer (0.2% of Triton X-100, 20 mM NH_4_OH in DMEM) was added to the cells and observed under a tissue culture microscope until the VSMCs were completely lysed (5–10 minutes). Total cell lysis was tested by analyzing 4′,6-diamidino-2-phenylindole staining in a coverslip. Then, control VSMCs were seeded in the ECMs at 100% of confluence and the experiments were analyzed after 24 hours.

### Real-Time and Quantitative PCR

Aortas were extracted after perfusion with 5 mL saline solution, and the adventitia layer was discarded. Liquid nitrogen frozen tissue was homogenized using a cold mortar and an automatic bead homogenizer (MagNA Lyzer, Roche). Total RNA was isolated with Trizol (Life Technologies). Total RNA (1 μg) was first digested with DNAse and reverse-transcribed with Maxima First Strand cDNA Synthesis Kit (ThermoFisher). For analysis of mtDNA levels, total DNA from cells and tissues was extracted with the SurePrep kit (Fisher Scientific) or Trizol, respectively, according to the manufacture’s guidelines. DNA was amplified using primers specific for cytochrome mt-Co1 (mitochondrially-encoded cytochrome *c* oxidase 1) and mitochondrial 16S rRNA, then normalized to *B2M* (β-2 macroglobulin) and *H2K* nuclear-encoded gene controls. Real-time quantitative PCR (qPCR) was performed with the primers indicated in Table I in the Data Supplement. qPCR reactions were performed in triplicate with SYBR master mix (Promega), according to the manufacturer’s guidelines. To examine probe specificity, we conducted a postamplification melting curve analysis. For each reaction, only 1 melting temperature peak was produced. The amount of target mRNA in samples was estimated by the 2−*C*T relative quantification method, using *B2M*, *YWHAZ* (tyrosine 3-monooxygenase/tryptophan 5-monooxygenase activation protein ζ), and *PP1A* (protein phosphatase 1 catalytic α) for normalization. Fold ratios were calculated relative to mRNA expression levels from controls.

### Library Preparation and Illumina Sequencing

Aortas were extracted after perfusion of cold saline solution and the adventitia layer was discarded. Frozen tissue was homogenized, and total RNA was isolated with Trizol (Roche). RNA from Libraries were prepared according to the instructions of the NEBNext Ultra Directional RNA Library prep kit for Illumina (New England Biolabs), following the Poly(A) mRNA Magnetic Isolation Module protocol. The input yield of total RNA to start the protocol was >300 ng quantified by an Agilent 2100 Bioanalyzer using an RNA 6000 nano LabChip kit. The obtained libraries were validated and quantified by an Agilent 2100 Bioanalyzer using a DNA7500 LabChip kit and an equimolecular pool of libraries was titrated by qPCR using the KAPA SYBR FAST qPCR Kit for LightCycler 480 (Kapa BioSystems) and a reference standard for quantification. After processing on the Illumina HiSeq 2500 instrument, FastQ files were generated containing nucleotide data and quality scores for each position. RNA-sequencing reads were mapped to the *Mus musculus* reference genome, GRCm38.p6, using Hisat2 v2.1.0 software. Reads were then preprocessed with SAMtools v1.7 to transform Sequence Alignment/Map files into Binary Alignment/Map files and sorted. These files were used as an input for the HTSeq v0.6.1 package that produces a file containing the mapped reads per gene (as defined by the *Mus musculus*, GRCm38.96 version, gff file) for each sample. Once obtained HTSeq read counts, the Bioconductor RNA-sequencing workflow was followed to detect the expression differences of genes using the DESeq2 statistical package. Ingenuity pathway analysis was used to identify cellular biologic functions, gene clusters, and upstream regulators.

### Immunoblot

For Western blot analysis, cells were lysed at 4°C in radioimmunoprecipitation assay buffer containing protease and phosphatase inhibitors cocktail (Sigma). Proteins were separated by SDS-PAGE and transferred onto 0.45-µm pore size polyvinylidene fluoride membranes (Immobilon-P PVDF membrane; Millipore). Polyvinylidene fluoride membranes were blocked with TBS-T (50 mM Tris, 150 mM NaCl, and 0.1% Tween-20) containing 5% (wt/vol) milk. Membranes were incubated with primary antibodies diluted from 1/500 to 1/1000, followed by TBS-T washes and incubation with HRP (horseradish peroxidase)−conjugated secondary antibodies (GE Healthcare). The signal was visualized by enhanced chemiluminescence with Luminata Forte Western HRP Substrate (Millipore) and the ImageQuant LAS 4000 imaging system. The following antibodies were used: anti-TFAM (Proteintech), anti-MT-CO1 (Millipore), anti-HIF1a and anti-mt-ND1 (Novus Biologicals), anti-P53 (Santa Cruz), anti-PGC1a (Thermo Scientific), anti-VDAC (Abcam), anti-Actin (Abcam), and anti–α-tubulin (Cell Signaling).

### Aortic Histology

After euthanization by CO_2_ inhalation, mice were perfused with saline. Aortas were then isolated and fixed in 10% formalin overnight at 4°C. Paraffin cross-sections (5 μm) from fixed organs were stained with Masson trichrome, Alcian blue, or Verhoeff Van Gieson elastic, or they were used for immunohistochemistry or immunofluorescence. Elastic fibers were stained with a modified Verhoeff Van Gieson elastin stain kit (Sigma-Aldrich). Elastic lamina breaks, defined as interruptions in the elastic fibers, were counted in the entire medial layer of 3 consecutive cross-sections per mouse, using 4 to 16 mice per experiment, and the mean number of breaks was calculated. For immunostaining, the deparaffinized sections were rehydrated, boiled to retrieve antigens (10 mM citrate buffer, 0.05%Triton X-100, pH 6) and blocked for 45 minutes with 10% goat normal serum, 5% horse serum, 0.05% TritonX-100, and 2% BSA in PBS. Samples were incubated with the following antibodies for immunohistochemistry or immunofluorescence: monoclonal anti-SMA (1/500, C6198; Sigma), polyclonal anti-TFAM (1/300; Proteintech), polyclonal anti-MT-ND1 (1/300; Proteintech), monoclonal anti-MT-CO1 (1/300; Invitrogen), polyclonal anti-HIF1A, and anti-MYC (1/500; Novus Biologicals). Specificity was determined by substituting primary antibody with unrelated IgG (Santa Cruz). For immunohistochemistry, endogenous peroxidase and biotin were blocked with 1% hydrogen peroxide−methanol for 10 minutes and a biotin blocking kit (Vector Laboratories), respectively. Color was developed in all samples at the same time with 3, 3-diaminobenzidine (DAB, Vector Laboratories), and sections were counterstained with hematoxylin and mounted in DPX (Fluka). For immunofluorescence, secondary antibodies were Alexa Fluor 546−conjugated goat anti-rabbit and Alexa Fluor 647−conjugated goat anti-rabbit (BD Pharmingen). For F-actin determination, fixed aortas were embedded in OCT (Tissue-Tek Sakura), and 5-μm cross-sections were incubated for 30 minutes with 1:1000 Phalloidin-657 (Millipore) after 10 minutes of incubation with 0.3% Triton X-100 in PBS. Sections were mounted with 4′,6-diamidino-2-phenylindole in Citifluor AF4 mounting medium (Aname). Images were acquired at 1024×1024 pixels and 8 bits using a Zeiss LSM800 microscope with 40× oil-immersion objectives.

### Vascular Reactivity

Aorta and mesenteric resistance arteries were dissected and 2-mm length segments were mounted in a wire myograph for isometric tension recording. After a 30-minute equilibration period in oxygenated Krebs−Henseleit solution at 37°C and pH 7.4, segments were stretched to their optimal lumen diameter for active tension development. Contractility of the segments was tested by an initial exposure to a high K+ solution (120 mmol/L). After washing, a concentration−response curve to increasing concentration of phenylephrine (1 nmol/L–50 µmol/L) and to U46619 (0.1 nmol/L–1 µmol/L) were carried out.

### Gelatin Zymography

Supernatants form cell culture were prepared as described.^32^ Extracts (15 μg) were fractioned under nonreducing conditions on 10% SDS–polyacrylamide gels containing 1% gelatin (Sigma). Gels were washed 3 times in 2.5% Triton X-100 for 2 hours at room temperature, incubated overnight at 37°C in 50 mM Tris-HCl (pH 7.5), 10 mM CaCl_2_, and 200 mM NaCl, and stained with Coomassie blue. The areas of gelatinolytic matrix metalloproteinase (MMP) activity were visualized as transparent bands. Images were analyzed with Quantity One software (Bio-Rad).

### Human Samples

The study was approved by the Ethics and Clinical Research Committee of Instituto de Salud Carlos III and Cantabria University (B2017/BMD-3676, AORTASANA-CM, ref. 27/2013; respectively). Aortas for use as controls were obtained anonymously from multiorgan transplant donors after written informed consent was obtained from their families. During preparation of the heart for transplantation, excess ascending aortic tissue was harvested for the study. Samples from patients were obtained during elective or emergency aortic root surgery for aortic aneurysm dissection. Patient clinical data were retrieved while maintaining anonymity. Tissues were immediately fixed, kept at room temperature for 48 hours, and embedded in paraffin. DNA and RNA extraction from paraffin sections were performed with All Prep DNA/RNA FFPE [formalin-fixed, paraffin-embedded] kit (Qiagen).

### Statistical Analysis

Normality of data were checked using a Shapiro–Wilk test. F-test was used to assess the equality of variance assumption. Differences between 2 groups were assessed using the unpaired Student *t* test, *t* test with Welch correction for unequal variances, or Mann–Whitney U test, where appropriate. Differences in experiments with ≥3 groups were analyzed by 1-way, 2-way, or repeated-measurement 2-way ANOVA, or a mixed-effects linear model and Newman post hoc test, as appropriate. For the statistical analysis of RNA-sequencing data, the *P* values were corrected using the false discovery rate method (false discovery rate <0.05). For survival curves, differences were analyzed with the log-rank (Mantel–Cox) test. Multiple linear regression analysis adjusted for age was performed with R software. For all other analysis GraphPad Prism software 9 was used. Statistical significance was indicated as *P<0.05, **P<0.01, ***P<0.001, and ****P<0.0001. Sample size was chosen empirically based on our previous experiences in the calculation of experimental variability; no statistical method was used to predetermine sample size. Before data analysis, outliers were identified and excluded by using the Robust Outlier removal method (Q value =5%) to identify outliers provided with GraphPad Prism 9.

The numbers of animals used are described in the corresponding figure legends. All experiments were done with at least 3 biologic replicates. Experimental groups were balanced in terms of animal age, sex, and weight. Animals were genotyped before experiments, and were all caged together and treated in the same way. Sex and age are indicated in the figure legends. Appropriate tests were chosen according to the data distribution. Variance was comparable between groups in experiments described throughout the article. For the rest of experiments, no randomization was used to allocate animals to experimental groups, and investigators were not blinded to group allocation during experiments or to outcome assessments.

## Results

### Aortas From Marfan Syndrome Mice Present Features of Mitochondrial Decline

**Figure 1.**
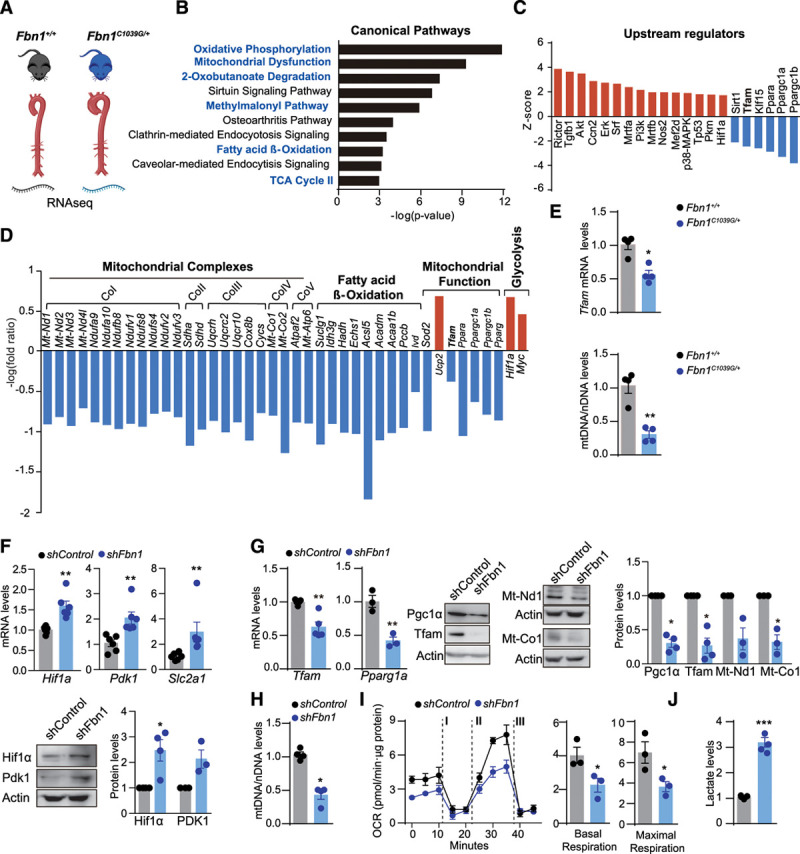
**Mitochondrial function decline in a mouse model of Marfan syndrome.** **A** through **D**, RNA-sequencing analysis of aortas from 4 *Fbn1*^*C1039G/+*^ mice with 3 *Fbn1*^+/+^ littermates (24-week-old male mice). **B**, Top 10 significantly changed canonical pathways predicted by ingenuity pathway analysis based on differentially regulated genes. Metabolism-related pathways are highlighted in blue lettering; *P*<0.01. **C**, Activation or inhibition of upstream regulators predicted by ingenuity pathway analysis (–2>bias-corrected z-score>4; *P*<0.05). The predicted inhibition of Tfam is highlighted. **D**, Expression of genes encoding mitochondrial complexes (Co) and fatty acid oxidation enzymes, and genes related to mitochondrial function and glycolysis; *P* < 0.05. **E**, Quantitative reverse transcription polymerase chain reaction analysis of *Tfam* mRNA expression and quantitative polymerase chain reaction analysis of relative mtDNA content in aortic extracts from 20-week-old *Fbn1*^*C1039G/+*^ and *Fbn1*^+/+^ male mice. **F** through **I**, Primary murine vascular smooth muscle cells transduced with *shFbn1* or *shControl* for 5 days. **F**, Quantitative reverse transcription polymerase chain reaction of *Hif1a*, *Pdk1*, and representative immunoblots analysis, and relative quantification of Hif1a and Pdk1 protein levels. **G**, Quantitative reverse transcription polymerase chain reaction of *Tfam and Ppargc1a* and representative immunoblot analysis and quantification of Pgc1α, Tfam, Mt-Nd1, and Mt-Co1. **H**, Quantitative polymerase chain reaction analysis of relative mtDNA content in *shFbn1*- and *shControl*-transduced vascular smooth muscle cells. **I**, OCR in *shFbn1* and *shControl* vascular smooth muscle cells at basal respiration and after addition of the complex V inhibitor oligomycin (I) and fluoro carbonyl cyanide phenylhydrazone (II) to measure maximal respiration, followed by a combination of rotenone and antimycin A (III). **J**, Levels of extracellular lactate in the supernatant from *shFbn1* and *shControl* vascular smooth muscle cells. Actin was used as total protein loading control. Data are mean±SEM Statistical significance was assessed by Student *t* test. *P<0.05, **P<0.01, ***P<0.001, ****P<0.0001 vs *Fbn1*^+/+^ mice (**E**), *shControl* (**F** through **J**). Acaa1b indicates 3-ketoacyl-CoA thiolase B, peroxisomal; Acsl5, Acyl-CoA Synthetase Long Chain Family Member 5; Akt, serine/threonine kinase 1; Atpaf2, ATP synthase mitochondrial F1 complex assembly factor 2; Ccn2, cellular communication network factor 2; CoI–V, mitochondrial complexes I-V; Cox8b, cytochrome c oxidase subunit 8B; Cycs, cytochrome c, somatic; Echs1, enoyl-CoA hydratase, short chain 1; Erk, extracellular signal regulated kinases; Fbn1, fibrillin-1; Hadh, hydroxyacyl-CoA dehydrogenase; Hif1a, hypoxia-inducible factor 1 α; Idh3g, isocitrate dehydrogenase (NAD(+)) 3 non-catalytic subunit gamma; Ivd, isovaleryl-CoA dehydrogenase; Mrtfa, b, myocardin related transcription factor A, B; Mt-Atp6, mitochondrially-encoded ATP synthase membrane subunit 6; Mt-Co1, 2, mitochondrially encoded cytochrome c oxidase I /II; Mt-Nd1–4I, mitochondrially encoded NADH dehydrogenase 1-4; Myc, Myc protoncogen, myelocytomatosis oncogene; Ndufa9, 10, NADH:ubiquinone oxidoreductase subunit A9-10; Ndufb8, NADH:ubiquinone oxidoreductase subunit B8; Ndufs4, 8, NADH:ubiquinone oxidoreductase core subunit S4,8; Ndufv1–3, NADH:ubiquinone oxidoreductase core subunit V1-3; Nos2, nitric oxide synthase 2; OCR, oxygen consumption rate; Mef2d, myocyte enhancer factor 2D; p38-MAPK, p38 mitogen-activated protein kinase; Pccb, propionyl-CoA carboxylase beta chain, mitochondrial; Pkm, Pyruvate kinase muscle isozyme; Pi3k, phosphatidylinositol 3-kinase; Pparg, Peroxisome proliferator-activated receptor gamma; Ppara, peroxisome proliferator-activated receptor α: Ppargc1a, b, peroxisome proliferator-activated receptor γ coactivators 1a and 1b, respectively; Rictor, RPTOR independent companion of MTOR, complex 2; Sdha, d, succinate dehydrogenase complex flavoprotein subunit A, D; shControl, Control Short hairpin RNA; shFbn1, Short hairpin RNA Fbn1; Sirt1, NAD-dependent deacetylase sirtuin-1; Slc2a1, Glut1, solute carrier family 2 member 1; Sod2, Superoxide dismutase 2, mitochondrial; Srf, serum response factor; Suclg1, succinate-CoA ligase GDP/ADP-forming subunit alpha; TCA, tricarboxylic acid cycle; Tfam, mitochondrial transcription factor A; Tgfb1, transforming growth factor β1; Tp53, tumor protein p53; Uqcr10, Ubiquinol-Cytochrome C Reductase, Complex III Subunit X; Ucp2, mitochondrial uncoupling protein 2; Uqcrc2, Ubiquinol-Cytochrome C Reductase Core Protein 2; and Uqcrh, Ubiquinol-Cytochrome C Reductase Hinge Protein.

*Fbn1*^*C1039G/+*^ mice reproduce the aortic dilation, aneurysms, and histologic features of aortic medial degeneration found in MFS patients.^28^ To identify novel molecular mechanisms underlying TAA formation, we performed transcriptional analysis in aortas from 24-week-old *Fbn1*^*C1039G/+*^ mice, showing an intermediate stage of the aortic disease (Figure 1A).^34,35^ Ingenuity pathway analysis revealed that 6 of the 10 most altered canonical pathways were related to metabolism, including oxidative phosphorylation, mitochondrial dysfunction, fatty acid β-oxidation, and the tricarboxylic acid cycle (Figure 1B). Ingenuity pathway analysis prediction of activity of upstream regulators identified canonical MFS regulators such as inducible Nos2 (nitric oxide synthase) and Tgfb1 (transforming growth factor β1).^28,32^ This analysis pointed to increased activity of the glycolytic regulator Hif1α (hypoxia-inducible factor α) and reduced activity of several mitochondrial biogenesis and function regulators, including Tfam (Figure 1C). *Fbn1*^*C1039G/+*^ aortas showed reduced expression of all mitochondrial complex subunits, including mitochondria- and nuclear-encoded genes, as well as of genes related to fatty acid β-oxidation and mitochondrial biogenesis and function (*Tfam, Ppara* [peroxisome proliferator-activated receptor α], *Pparg* [peroxisome proliferator-activated receptor γ], *Ppargc1a* [PPARG coactivator 1α], *Ppargc1b* [PPARG coactivator 1β], and *Sod2* [superoxide dismutase 2, mitochondrial]*).* In contrast, genes involved in mitochondrial uncoupling (*Ucp2* [uncoupling protein 2]) and glycolytic rewiring (*Hif1a* [hypoxia-inducible factor-1α], *Myc*) were upregulated (Figure 1D). Quantitative reverse transcription PCR analysis confirmed reduced expression of *Tfam* (Figure 1E). Tfam is a nuclear-encoded mitochondrial transcription factor that controls the transcription, replication, and stability of mtDNA.^23^ Analysis of relative mtDNA content in *Fbn1*^*C1039G/+*^ aortas revealed below-normal mtDNA content (Figure 1E). To model MFS in vitro, we silenced *Fbn1* with lentiviral vectors in primary murine VSMCs (Figure IA in the Data Supplement). Confirming the transcriptomic data from MFS mice, *shFbn1* VSMCs displayed increased levels of Hif1α and its targets Pdk1 (pyruvate dehydrogenase kinase-1) and *Slc2a1* (glucose-transporter 1; Figure 1F), and significantly reduced expression of Pgc1α (*Ppargc1a*), as well as Tfam and its targets, MT-Nd1 (mitochondrially-encoded NADH-ubiquinone oxidoreductase chain 1) and MT-Co1 (Figure 1G), correlating with a reduction in mtDNA content (Figure 1H). Flux analysis of the OCR, an index of mitochondrial oxidative phosphorylation, revealed reduced mitochondrial respiration in Fbn1-silenced cells (Figure 1I). This was accompanied by increased production of extracellular lactate, an indicator of glycolysis (Figure 1J). As mitochondrial decline induces cellular senescence through p53,^36^ and our ingenuity pathway analysis predicts p53 (Tp53) as an active upstream regulator (Figure 1C), we analyzed senescence features in *shFbn1* VSMCs. Both protein and mRNA p53 levels were upregulated in *shFbn1* VSMCs (Figure IB in the Data Supplement) together with increased SABG (senescence-associated β-galactosidase) activity (Figure IC in the Data Supplement) and increased expression of proinflammatory cytokines related to SASPs (senescence-associated secretory phenotype) such as *Tnfa, Il1b* (interleukin 1β), and *Il6* (interleukin 6; Figure ID in the Data Supplement).

These data support that aortas from *Fbn1*^*C1039G/+*^ mice or VSMCs carrying MFS mutations present mitochondrial respiration decline, rewire their metabolism toward glycolysis, and display senescence and inflammation features.

### Mitochondrial Decline Appears in the Onset of Aortic Disease in *Fbn1*^*C1039G/+*^ Mice

**Figure 2.**
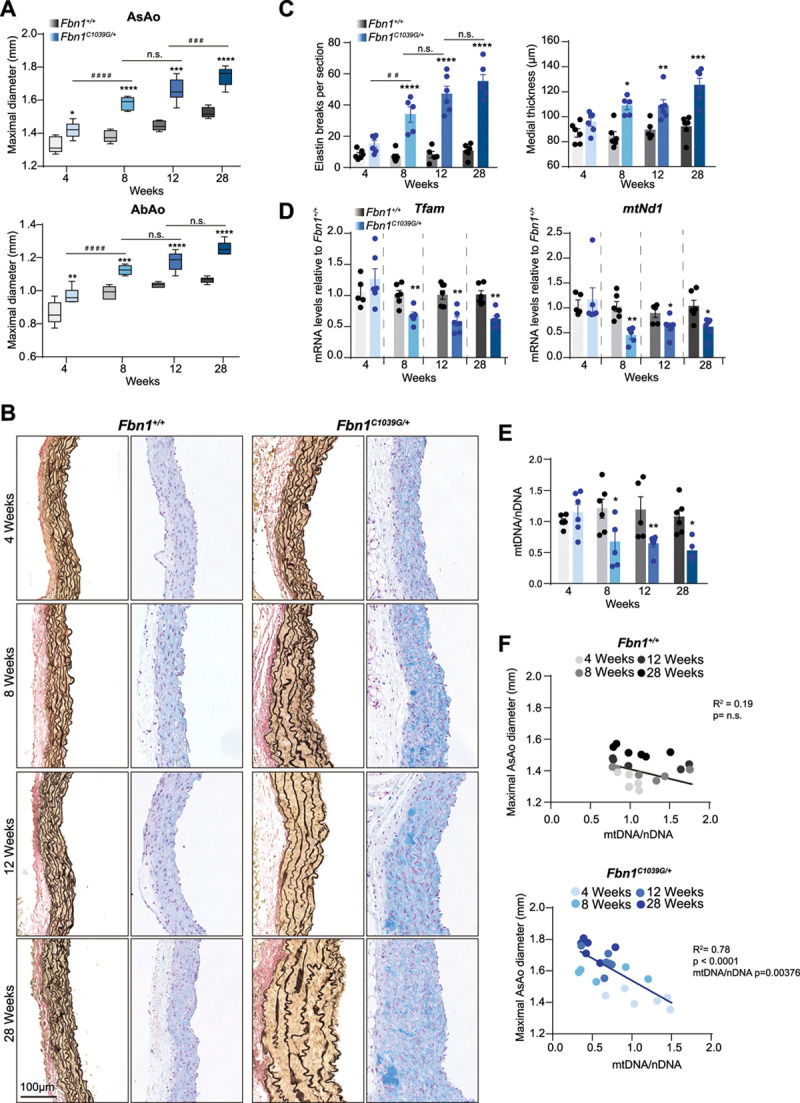
**Decrease of Tfam levels and mitochondrial DNA correlate with aortic deterioration of Fbn1C1039G/+ mice.** Analysis of the progression of mitochondrial and aortic phenotype in *Fbn1*^+/+^ and *Fbn1*^*C1039G/+*^ male mice from 4, 8, 12, and 28 weeks old. **A**, Maximal AsAo and AbAo diameter. **B**, Representative images of Van Gienson and Alcian blue histologic staining. **C**, Quantification of elastin breaks per section and aortic medial thickness in ascending aortas at the indicated ages. **D**, Relative quantitative reverse transcription polymerase chain reaction analysis of *Tfam* and *Mt-Co1* mRNA expression in aortic extracts from *Fbn1*^+/+^. **E**, Quantitative polymerase chain reaction analysis of relative mtDNA content in aortas from 4-week-old *Fbn1*^+/+^ mice and (**F**) multiple linear regression adjusted for age, between the AsAo diameter and mtDNA/nDNA levels; adjusted *R*^2^ and *P* value are indicated. Histograms show mean±SEM. Aortic diameter is presented in box and whisker plots showing maximal and minimal values and 75th and 25th percentiles. Statistical significance was assessed by 2-way ANOVA (**A**, **C**, **E**), Student *t* test (**D**), and multiple linear regression (**F**). *P<0.05, **P<0.01, ***P<0.001, ****P<0.0001 vs. *Fbn1*^+/+^ mice; ^##^*P*<0.01, ^###^*P*<0.001 vs. *Fbn1*^*C1039G/+*^ mice. AbAo indicates abdominal aorta; AsAo, ascending aorta; Fbn1, fibrillin-1; mtNd1, mitochondrially-encoded NADH ubiquinone oxidoreductase core subunit 1; n.s., not significant; and Tfam, mitochondrial transcription factor A.

To assess the stage during the aortic disease in which mitochondrial decline appears, we performed analysis of aortic diameter (Figure 2A) and histologic features (Figure 2B and 2C) alongside *Tfam* and mtDNA levels (Figure 2D and 2E) at different stages of TAA disease in *Fbn1*^*C1039G/+*^ mice. Aortas from 8-week-old mice already present significant below-normal *Tfam* mRNA levels, mtDNA content, and *Mt-Nd1* expression (Figure 2D) together with the appearance of a significant increase in elastin breaks, proteoglycan deposition, and aortic medial thickness (Figure 2B and 2C). Similarly, some well-known MFS aortopathy-related genes and metabolic genes already showed altered levels in aortas from 8-week-old *Fbn1*^C1039G/+^ mice (Figure II in the Data Supplement). In addition, the diameter of the aorta negatively correlated with mtDNA levels in MFS mice (Figure 2F).

Reduction in Tfam levels induces mtDNA leakage into the cytoplasm and activates the innate immune pathway stimulator of interferon genes (cGAS-STING).^24,37^ Interestingly, both *Irf7* (interferon regulatory factor 7) and *Isg15* (interferon-stimulated gene 15), which are cGAS-STING response target genes, were up-regulated in *Fbn1*^C1039G/+^ aortas at early stages of the disease (Figure II in the Data Supplement), supporting a previous report on the role of this route on aortic degeneration.^38^ Together these data suggest that mitochondrial decline appears with the first signs of aortic remodeling in *Fbn1*^*C1039G/+*^ mice.

### Decreased Tfam Expression and mtDNA Levels in Aortas from Human MFS Patients

**Figure 3.**
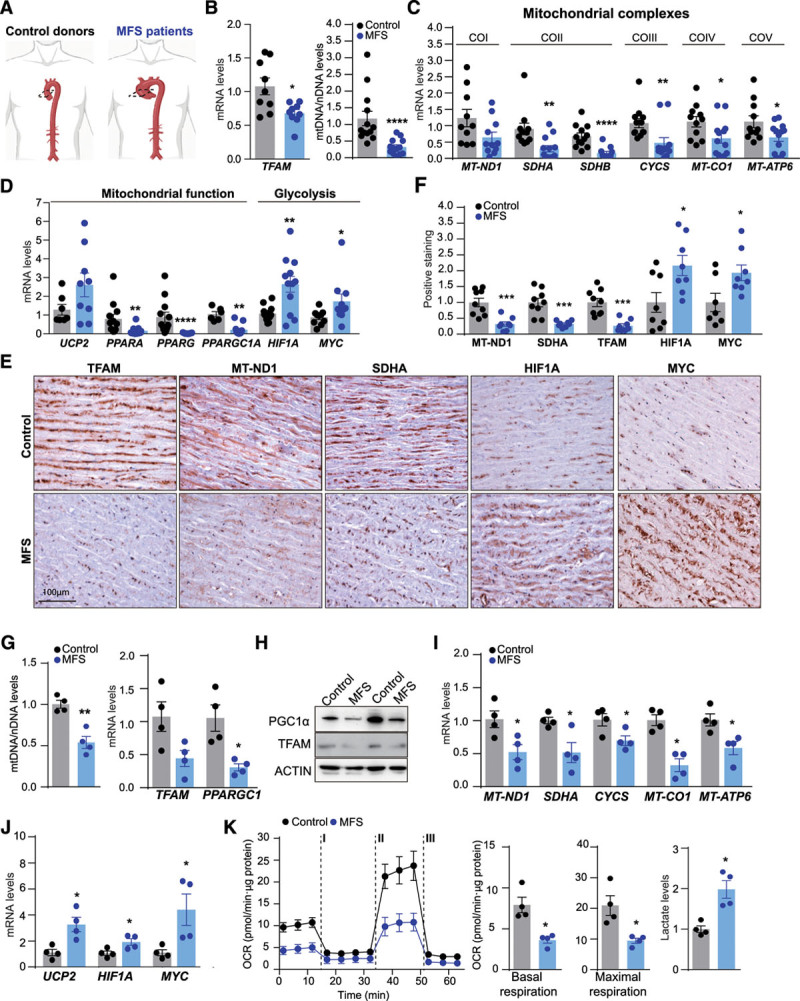
**Decrease of TFAM levels and mitochondrial respiration in samples from MFS patients.** **A**, Analysis of human ascending aortic samples from MFS patients and control donors. **B**, Quantitative reverse transcription polymerase chain reaction analysis of *TFAM* mRNA and quantitative polymerase chain reaction analysis of relative mtDNA content. **C**, Expression of genes encoding mitochondrial complex components and (**D**) genes related to mitochondrial function and glycolysis. **E**, Representative medial layer sections of immunohistochemical analysis of MT-ND1 (CoI), SDHA (CoII), TFAM, HIF1A, and MYC levels and (**F**) quantification. **G** through **K**, Primary fibroblasts from 4 patients with MFS and 4 healthy controls. **G**, Quantitative polymerase chain reaction analysis of mtDNA content and quantitative reverse transcription polymerase chain reaction analysis of *TFAM* and *PPARGC1A* expression (**H**) and representative TFAM and PGC1α immunoblotting (n=4). **I**, mRNA of *MT-ND1, SDHA, CYCS, MT-CO1*, *MT-ATP6*, (**J**) *UCP2, HIF1A*, and *MYC* as assessed by quantitative reverse transcription polymerase chain reaction, in extracts from human MFS and Control fibroblast. **K**, OCR after addition of oligomycin (I), fluoro carbonyl cyanide phenylhydrazone (II), and a combination of rotenone and antimycin-A (III) and extracellular lactate levels. Data are mean±SEM. Statistical significance was assessed by Student *t* test *P<0.05, **P<0.01, ***P<0.001, ****P<0.0001 vs Control. ACTIN indicates beta actin; COI–V, mitochondrial complexes I-V; CYCS, Cytochrome C, Somatic; HIF1A, hypoxia-inducible factor 1 α; MFS, Marfan syndrome; MT-ATP6, mitochondrially-encoded ATP synthase membrane subunit 6; MT-CO1, mitochondrially-encoded cytochrome *c* oxidase I; MT-ND1, mitochondrially-encoded NADH ubiquinone oxidoreductase core subunit 1; MYC, MYC proto-oncogene, bHLH transcription factor; PPARA, Peroxisome proliferator-activated receptor alpha; PPARAGC, peroxisome proliferator-activated receptor γ coactivator; PPARAGC1a, Peroxisome proliferator-activated receptor gamma coactivator 1-alpha; PGC1α, Peroxisome proliferator-activated receptor gamma coactivator 1-alpha (protein); SDHA, B, succinate dehydrogenase complex flavoprotein subunit A-B; TFAM, mitochondrial transcription factor A; and UCP2, mitochondrial uncoupling protein 2.

To assess whether the same metabolic rewiring occurs in aortas from MFS patients (Figure 3A), we measured TFAM mRNA and mtDNA levels and the expression of mitochondrial function genes in TAAs from MFS patients. Compared with healthy control samples, TAA samples from MFS patients showed significantly lower levels of mRNA of TFAM and mtDNA (Figure 3B), as well as reduced transcription of genes that encode mitochondrial complexes *(MT-ND1, SDHA* [succinate dehydrogenase complex, subunit A], *SDHB* [succinate dehydrogenase complex, subunit B], *CYCS* [cytochrome complex], *MT-CO1*, and *MT-ATP6* [mitochondrially-encoded ATP synthase membrane subunit 6]) or are involved in mitochondrial function (*PPARA, PPARG*, and *PPARG1A*; Figure 3C and 3D). Moreover, aneurysm samples showed upregulation of genes involved in mitochondrial uncoupling and glycolytic rewiring (*UCP2, HIF1A*, and *MYC*; Figure 3D). Histologic analysis of TFAM, the mitochondrial proteins MT-ND1, SDHA and the glycolysis-related genes HIF1A and MYC supported glycolytic rewiring in MFS aortic samples (Figure 3E and 3F). Coimmunostaining for smooth muscle actin confirmed that mitochondrial gene down-regulation was specific to VSMCs (Figure III in the Data Supplement). Moreover, we used primary skin fibroblasts from 4 individuals with MFS and 4 healthy donors to analyze the mitochondrial respiratory capacity in vitro. MFS fibroblast presented a reduction in the total mtDNA content, *TFAM* and *PPARGC1a* mRNA, and protein expression (Figure 3G and 3H). In line with human MFS aortas, primary MFS skin fibroblasts showed a decrease of the mRNA levels of *MT-ND1, SDHA, MT-CO1*, and *MT-AP6* and increased expression of *UCP2, HIF1A*, and *MYC* (Figure 3I and 3J). Moreover, MFS fibroblasts showed a reduction in the OCR and increased lactate production (Figure 3K). Altogether, these data support that aortas from MFS present metabolic rewiring toward glycolysis because of mitochondrial function decline.

### Extracellular Regulation of Mitochondrial Respiration in MFS

**Figure 4.**
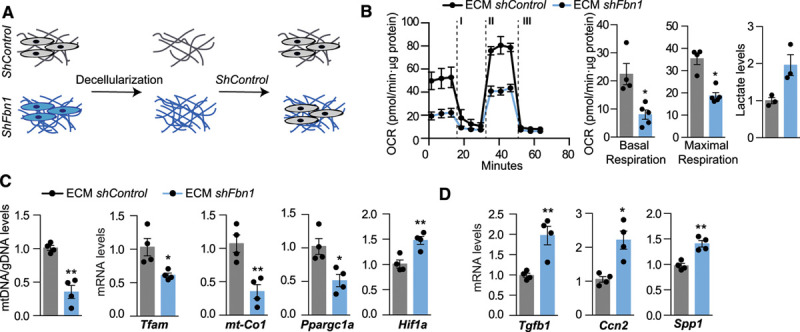
**ECM derived from thoracic aortic aneurysm cells decreases Tfam levels and mitochondrial respiration.** **A**, Primary vascular smooth muscle cells transduced with *shFbn1* or *shControl* were cultured for 5 days, lead to produce ECM, then the matrices were decellularized and *shControl* cells were seeded. **B**, OCR in *shControl* vascular smooth muscle cells seeded in *shControl* and *ShFbn1*-ECM at basal respiration and after addition of oligomycin (I) and fluoro carbonyl cyanide phenylhydrazone (II) to measure maximal respiration, followed by a combination of rotenone and antimycin A (III) and extracellular lactate levels. **C**, Quantitative reverse transcription polymerase chain reaction analysis of *Tfam, Mt-co1, Ppargc1, and Hif1a*, and quantitative polymerase chain reaction analysis of mtDNA content. **D**, Quantitative reverse transcription polymerase chain reaction analysis of synthetic genes *Tgfb1, Ccn2*, and *Spp1*. Statistical significance was assessed by Student *t* test. *P<0.05, **P<0.01 vs Control. Ccn2 indicates cellular communication network factor 2; ECM, extracellular matrix; Hif1a, hypoxia-inducible factor 1 α; mt-Co1, mitochondrially-encoded cytochrome *c* oxidase I; OCR, oxygen consumption rate; Ppargc1, peroxisome proliferator-activated receptor γ coactivator 1; shFbn1, Short hairpin RNA Fbn1; Spp1, secreted phosphoprotein 1; Tfam, mitochondrial transcription factor A; and Tgfb1, transforming growth factor β1;

Because elevated arterial stiffness is a characteristic of TAA,^16,17,39^ and recent evidence supports that ECM stiffness and composition affect cellular metabolism,^22^ we hypothesized that VSMC metabolism could be regulated by the microenvironment. To investigate this hypothesis, we transferred control VSMCs to plates coated with ECM-produced by Fbn1-deficient VSMCs (Figure 4A). Importantly, ECM from TAA cells induced a reduction in the OCR and increased lactate production in control VSMCs (Figure 4B). In addition, TAA ECM induced a decrease in the levels of *Tfam* mRNA and in the mtDNA content and reduced the transcription of the mitochondrial genes *Mt-Co1* and *Ppargc1a* while increasing the transcription of the glycolytic transcription factor *Hif1a* (Figure 4C). Moreover, control VSMCs cultured on ECM produced by Fbn1-deficient VSMCs up-regulated the expression of synthetic genes (*Tgfb1, Spp1* [osteopontin], *andCcn2* [cellular communication network factor 2]; Figure 4D), suggesting that both the metabolism and the phenotype of VSMCs depends on signals coming from the extracellular matrix.

### Mitochondrial Dysfunction in VSMCs Causes Aortic Aneurysm

**Figure 5.**
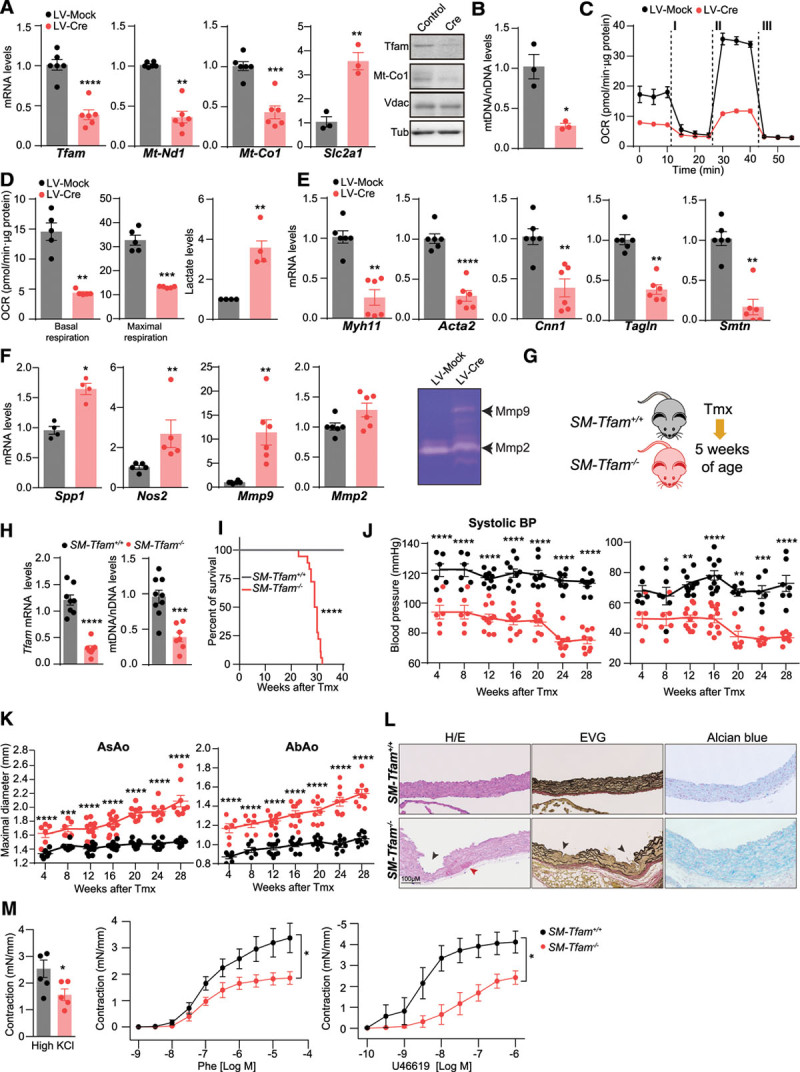
**Ablation of Tfam in vascular smooth muscle cells induces synthetic phenotype and aortic remodeling.** **A** through **F**, Primary mouse Tfam^flox/flox^ vascular smooth muscle cells were transduced with LV-Mock or LV-Cre lentivectors and analyzed after 10 days. **A**, Quantitative reverse transcription polymerase chain reaction analysis of relative *Tfam*, and RT *Mt-Nd1, Mt-Co1*, and *Slc2a1* mRNA expression and representative immunoblot analysis of Tfam and Mt-Co1; Vdac and Tub were used as mitochondrial and total protein loading controls, respectively. **B**, Quantitative polymerase chain reaction analysis of relative mtDNA content. **C**, OCR at (**D**) basal respiration and after addition of oligomycin (I) and fluoro carbonyl cyanide phenylhydrazone (II) to measure maximal respiration, followed by a combination of rotenone and antimycin A (III); and normalized extracellular lactate levels. **E**, Quantitative reverse transcription polymerase chain reaction assessed relative mRNA expression of the smooth muscle contractile genes *Myh11*, *Acta2*, *Cnn1, Tagln*, and *Smtn*. **F**, Quantitative reverse transcription polymerase chain reaction assessed relative mRNA expression of the vascular smooth muscle cells synthetic phenotype genes *Spp1, Nos2, Mmp9*, and *Mmp2*. **F**, **right**, Representative gelatin zymograph from 24 h conditioned medium, indicating Mmp9 and Mmp2 enzymatic activity. **G**, Experimental design for **H** through **L**: *SM-Tfam*^+/+^ and *SM-Tfam*^−/−^ mice were treated with tamoxifen at 5 weeks old. **H**, Quantitative reverse transcription polymerase chain reaction analysis of *Tfam* expression and quantitative polymerase chain reaction analysis of mtDNA content in aortic extracts from *SM-Tfam*^+/+^ and *SM-Tfam*^−/−^ mice 28 weeks after tamoxifen injections. **I**, Percent survival after tamoxifen injections in *SM-Tfam*^+/+^ and *SM-Tfam*^−/−^ mice; n=20. **J**, Evolution of systolic and diastolic blood pressure after tamoxifen injections in *SM-Tfam*^+/+^ and *SM-Tfam*^−/−^ mice. **K**, Evolution of maximal ascending aorta and abdominal aorta diameters after tamoxifen treatment in *SM-Tfam*^+/+^ and *SM-Tfam*^−/−^ mice. **L**, Representative images of histologic staining with H/E, EVG, and Alcian blue in ascending aortas from *SM-Tfam*^+/+^ and *SM-Tfam*^−/−^ mice 28 weeks after tamoxifen injection. Black arrowheads indicate aortic dissections; red arrowheads indicate intramural hematomas; n=4. **M**, Aortic contractile responses to high KCl solution and concentration–response curves to Phe and U46619; n=5. Data are mean±SEM. Statistical significance was assessed by Student *t* test (**A** through **H**), log-rank (Mantel–Cox) test (**I**), mixed-effects linear model (**J**, **K**), or 2-way ANOVA repeated measures (**M**, **right**). *P<0.05, **P<0.01, ***P<0.001, ****P<0.0001 vs Lv-Mock (**A** through **F**) or vs *SM-Tfam*^+/+^ mice (**H** through **M**). AbAo indicates abdominal aorta; Acta2, actin α2, smooth muscle; AsAo, ascending aorta; Bp, blood pressure; Cnn1, calponin 1; Cre, Cre recombinase; EVG, elastin Van Gienson; H/E, hematoxylin-eosin; LV-Cre, Cre-expressing; LV-Mock, green fluorescent protein–expressing; Mmp2, 9, matrix metalloproteinase 2 and 9, respectively; Mt-Co1, mitochondrially-encoded cytochrome *c* oxidase I; Mt-Nd1, mitochondrially-encoded NADH ubiquinone oxidoreductase core subunit 1; Myh11, myosin heavy chain 11; Nos2, nitric oxide synthase 2; OCR, oxygen consumption rate; Phe, phenylephrine; Slc2a1, solute carrier family 2 member 1; *SM-Tfam*^+/+^, tamoxifen–treated *Myh11-Cre*^*ERT2*^*Tfam*^*Wt/Wt*^ mice; *SM-Tfam*^−/−^, tamoxifen–treated *Myh11-Cre*^*ERT2*^*Tfam*^*flox/flox*^ mice; Smtn, smoothelin; Spp1, secreted phosphoprotein 1; Tagln, transgelin; Tfam, mitochondrial transcription factor A; Tmx, tamoxifen; Tub, tubulin; U46619, U46619 synthetic analog of the prostaglandin PGH2; and Vdac, Voltage-dependent anion channel mitochondrial.

To investigate whether the mitochondrial decline plays a role in VSMC function and in the progression of aortic diseases, we induced mitochondrial dysfunction in VSMCs by targeting Tfam. For in vitro experiments, we transduced aortic VSMCs from *Tfam*^*flox/flox*^ mice with lentiviral vectors encoding the Cre recombinase. *Tfam* deletion was confirmed by analyzing *Tfam* mRNA and protein levels (Figure 5A). In accordance with its role in regulating mtDNA levels, Tfam deletion in VSMCs led to a reduction in mtDNA content that correlated with a decreased expression of the mtDNA-encoded genes *Mt-Co1* and *Mt-Nd1* and a reduced OCR, alongside with an increase in *Slc2a1* expression and lactate production (Figure 5A–5D). We also observed a sharp decrease in the mRNA expression of genes involved in contractility, such as smooth muscle *Myh11*, *Acta2*, *Tagln* (transgelin), *Cnn1* (calponin), and *Smtn* (smoothelin; Figure 5E). Moreover, this was accompanied by increased mRNA expression of genes related to the secretory phenotype, such as *Spp1*, the metalloproteinases *Mmp2* and *Mmp9*, and the inducible *Nos2* (Figure 5F). The increased *Mmp9* expression was accompanied by a marked increase of Mmp9 enzymatic activity in cell supernatants (Figure 5F). As observed in Fbn-1–deficient VSMCs, Tfam-depleted VSMCs showed an increase of p53 expression, senescent-associated β-galactosidase activity, and the proinflammatory cytokines *Tnfa* and *Il6* mRNA levels (Figure IV in the Data Supplement).

To analyze the effect of Tfam deletion in VSMCs in vivo, we crossed *Tfam*^*flox/flox*^ mice^23^ with mice expressing the Cre^ERT2^ fusion protein under the smooth muscle myosin (*Myh11*) promoter (*Myh11-Cre*^*ERT2*^^29^; Figure 5G). Analysis 28 weeks after tamoxifen treatment confirmed efficient abrogation of *Tfam* expression in aortas from *Myh11-Cre*^*ERT2*^*Tfam*^*flox/flox*^ mice, but not in tamoxifen–treated *Myh11-Cre*^*ERT2*^*Tfam*^*Wt/Wt*^ (hereafter referred to as *SM-Tfam*^−/−^ and *SM-Tfam*^+/+^ mice, respectively; Figure 5H). *Tfam* deletion was accompanied by a decrease in mtDNA content (Figure 5H). Lifespan analysis showed a significant decrease in survival rate, with 100% of *SM-Tfam*^−/−^ mice dying before 33 weeks after tamoxifen (Figure 5I). Longitudinal analysis of vascular phenotype revealed a decrease in blood pressure alongside with an increase in aortic diameter (Figure 5J and 5K). Histologic analysis of *SM-Tfam*^−/−^ mice 28 weeks after tamoxifen revealed aortic dissections, intramural hematomas, and medial degeneration, with elastin lamina degradation and proteoglycan accumulation in the ascending aorta (Figure 5L). Aortas from *SM-Tfam*^−/−^ mice also showed defective vascular contractility responses to high K^+^ solution, the α1-adrenergic agonist phenylephrine, and the thromboxane receptor A_2_ agonist U46619 (Figure 5M). Similar to MFS, aortas from *SM-Tfam*^−/−^ mice showed an increase of the cGAS-STING pathway genes (*Irf7*, *Isg15*, *Tmem173* [transmembrane protein 173], *Mb21d1* [mab-21 domain-containing protein 1]), proinflammatory cytokines, such as *Il1b, Tnfa*, and *Il6*, and the senescent marker p21 *(Cdkn1a*; Figure IVC in the Data Supplement). Consistently, Tfam deficiency in VSMCs greatly affects the vascular contractility and function.

**Figure 6.**
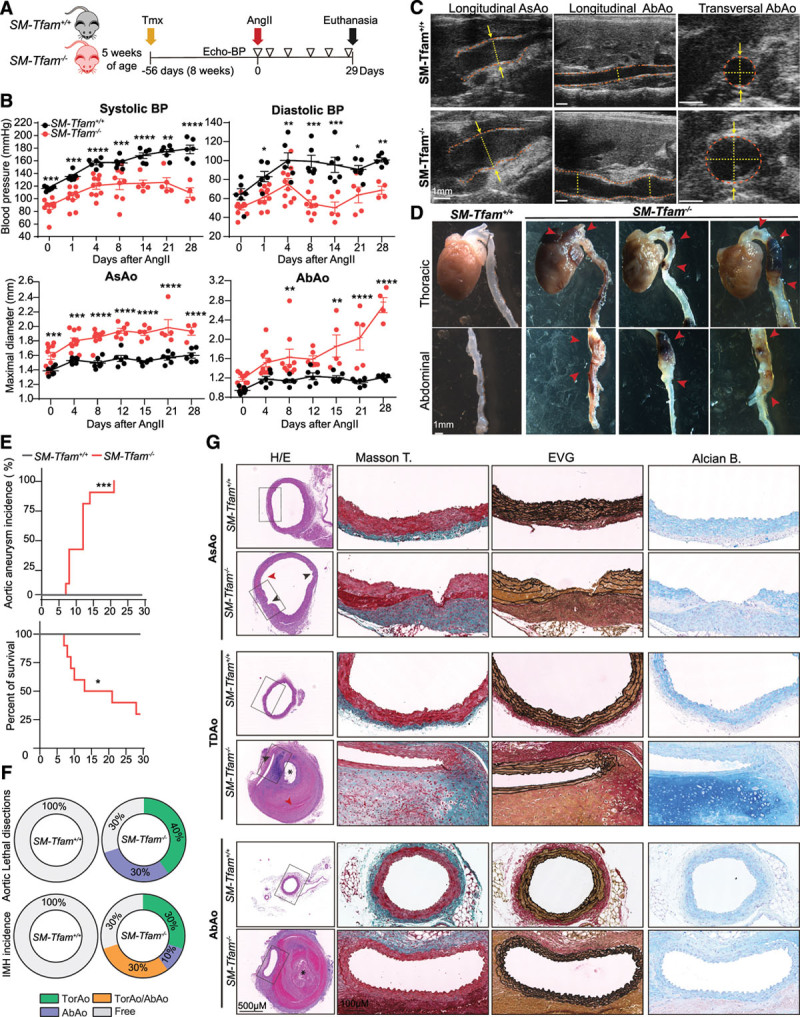
**Infusion of Ang II in SM-Tfam^−/−^ mice predisposes mice to lethal aortic aneurysm and dissections.** **A**, Experimental design: 56 days (8 weeks) after tamoxifen injections, Ang II minipumps were implanted in 6 *SM-Tfam*^+/+^ and 10 *SM-Tfam*^−/−^ male mice. Ultrasound and blood pressure (BP) analysis was performed 6 times (empty triangles). **B**, Evolution of systolic and diastolic BP (top) and maximal AsAo and AbAo diameters (bottom) on Ang II infusion. **C**, Representative aortic ultrasound images after 28 days of Ang II infusion in *SM-Tfam*^+/+^ and *SM-Tfam*^−/−^ mice. Discontinuous red lines mark the lumen boundary and discontinuous yellow lines and arrows denote the lumen diameter. **D**, Representative macroscopic images of aortas from the same animal cohort shown in **A**. Red arrowheads indicate aneurysms, dissections, and intramural hematomas. **E**, Aortic aneurysm incidence and percent survival of Ang II−infused *SM-Tfam*^+/+^ and *SM-Tfam*^−/−^ mice from the same cohort shown in **A**. **F**, Incidence and localization of lethal aortic dissections and IMH in the same cohort shown in **A**. **G**, Representative histologic analysis on sections of AsAo, TDAo, and AbAo from the same cohort shown in **A**. Statistical significance was assessed by mixed-effects linear model (**B**) and log-rank (Mantel–Cox) test (**E**). Data are mean±SEM. Red arrows indicate intramural hematomas. *False lumen; *P*<0.05, **P<0.01, ***P<0.001, ****P<0.0001 vs. *SM-Tfam*^+/+^ mice. AbAo indicates abdominal aorta; Alcian B, Alcian blue; AngII, angiotensin II; AsAo, ascending aorta; BP, blood pressure; EVG, elastin Van Gienson; H/E, hematoxylin eosin; IMH, intramural hematomas; Masson T, Masson’s trichrome; MFS, Marfan syndrome; NR, nicotinamide riboside; *SM-Tfam*^+/+^, tamoxifen–treated *Myh11-Cre*^*ERT2*^*Tfam*^*Wt/Wt*^ mice; *SM-Tfam*^−/−^, tamoxifen–treated *Myh11-Cre*^*ERT2*^*Tfam*^*flox/flox*^ mice; TDAo, thoracic descending aorta; Tfam, mitochondrial transcription factor A; Tmx, tamoxifen; TorAo, Thoracic aorta; and TorAo/AbAo, Thoracic/Abdominal aorta.

To test how *SM-Tfam*^−/−^ mice respond to a hypertensive challenge, we infused *SM-Tfam*^+/+^ and *SM-Tfam*^−/−^ mice with Ang II 56 days after tamoxifen (Figure 6A). Analysis of blood pressure showed a modest increase in *SM-Tfam*^−/−^ mice (Figure 6B) compared with *SM-Tfam*^+/+^. Ultrasonography revealed a rapid increase in the diameter of the ascending and abdominal aortas of *SM-Tfam* ^−/−^ mice, supporting the predisposition of these mice to development of aortic aneurysms (Figure 6B–6E). Most importantly, treatment of *SM-Tfam*^−/−^ mice with Ang II triggered aortic aneurysms and lethal aortic dissections, reducing mean survival (Figure 6E). Postmortem analysis revealed the presence of intramural hematomas and aortic ruptures with hemothorax or hemoabdomen (Figure 6F). Histologic analysis of thoracic and abdominal aortic sections showed increased aortic diameter, aortic dissections, intramural hematomas, false lumen formation, and features of medial degeneration, including elastic fiber fragmentation and disorganization, medial thickening, and accumulation of proteoglycans (Figure 6G). These data support that mitochondrial function in VSMCs is an important regulator of aortic function, and demonstrate that mitochondrial dysfunction in VSMCs induces aortic aneurysm and lethal dissections.

### NR Normalizes Mitochondrial Respiration in VSMCs Carrying TAA Mutations

**Figure 7.**
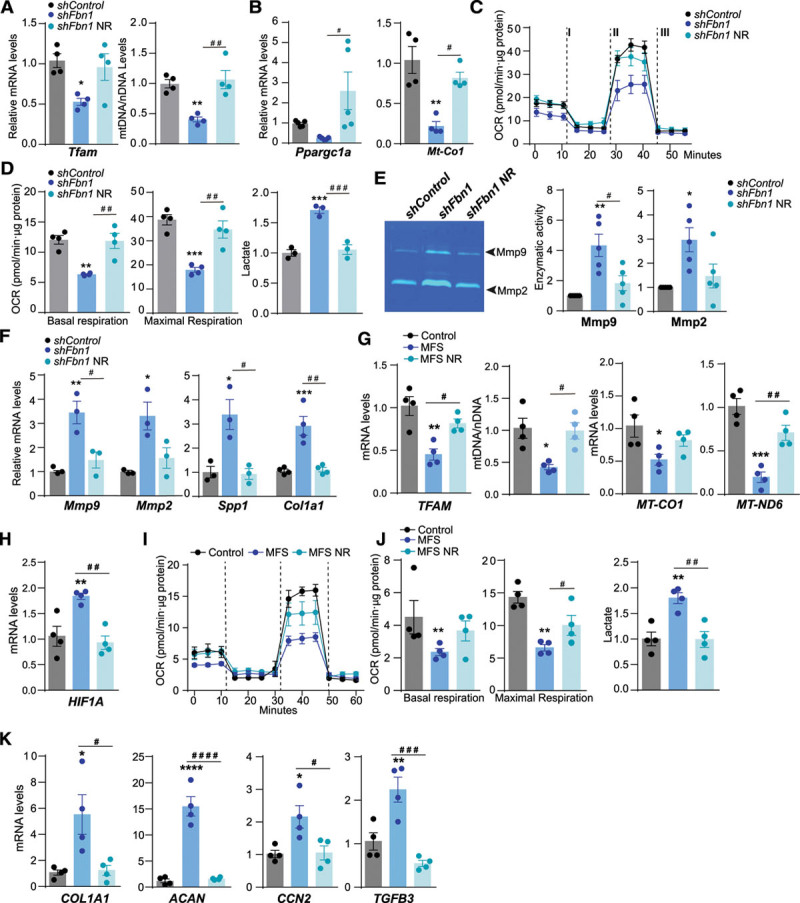
**NR increases Tfam levels and mitochondrial respiration in murine and human MFS cells.** **A** through **F**, Primary vascular smooth muscle cells transduced with *shFbn1* or *shControl* were treated with NR for 5 days. **A**, Quantitative reverse transcription polymerase chain reaction (RT-qPCR) analysis of *Tfam* and quantitative polymerase chain reaction analysis of mtDNA content. **B**, RT-qPCR analysis of *Ppargc1a* and *Mt-Co1* mRNA expression. **C** through **D**, OCR in *shFbn1* and *shControl* vascular smooth muscle cells after incubation with or without NR at basal respiration, and after addition of oligomycin (I) and fluoro carbonyl cyanide phenylhydrazone (II) to measure maximal respiration, followed by a combination of rotenone and antimycin A (III) and extracellular lactate levels. **E**, Representative gelatin zymogram images (left) and enzymatic activity analysis of Mmp2 and Mmp9 in 24h conditional medium (right). **F**, RT-qPCR analysis of *Mmp9, Mmp2, Spp1*, and *Col1a1* mRNA expression. **G** through **K**, Effect of NR on primary dermal fibroblasts from 4 MFS patients and 4 healthy donors (Control); cells were treated with NR for 5 days. **G**, RT-qPCR analysis of TFAM mRNA expression, quantitative polymerase chain reaction analysis of mtDNA content, and RT-qPCR analysis of *MT-CO1, MT-ND6, TFAM*, and (**H**) *HIF1A* mRNA expression. **I**, OCR after addition of oligomycin (I), fluoro carbonyl cyanide phenylhydrazone (II), and a combination of rotenone and antimycin A (III). **J**, Basal and maximal respiration rate and extracellular lactate levels. **K**, RT-qPCR analysis of *COL1A1, ACAN, CCN2*, and *TGFB3* mRNA expression. Data are mean±SEM. Statistical significance was assessed by 1-way ANOVA: *P<0.05, **P<0.01, ***P<0.001, ****P<0.0001 vs *ShControl* or Control; # *P*<0.05, ## *P*<0.01, ###*P*<0.001, ####*P*<0.0001 vs *ShFbn1* or MFS NR. ACAN indicates aggrecan; CCN2, cellular communication network factor 2; COL1A1, collagen type I α1 chain; HIF1A, hypoxia-inducible factor 1 α; MFS, Marfan syndrome; Mmp2, 9, matrix metalloproteinases 2 and 9, respectively; MT-CO1, mitochondrially-encoded cytochrome *c* oxidase I; MT-ND6, mitochondrially-encoded NADH-ubiquinone oxidoreductase chain 6; NR, nicotinamide riboside; OCR, oxygen consumption rate; Ppargc1a, peroxisome proliferator-activated receptor γ coactivator 1a; Spp1, secreted phosphoprotein 1; TFAM, mitochondrial transcription factor A; and TGFB3, transforming growth factor β3.

NAD^+^ is a cofactor for several enzymes with a critical role in the maintenance of cellular metabolism and mitochondrial function.^40^ NAD^+^ precursor supplementation has been proposed as a strategy to improve mitochondrial function conditions related to mitochondrial decline,^41–43^ including senescence.^44^ NR is a NAD precursor that improves mitochondrial metabolism by increasing Pgc1α and Tfam expression through sirtuin activity.^42,45^ Importantly, cellular production of NAD^+^ via Nampt (nicotinamide phosphoribosyltransferase) protects against DNA damage and premature senescence in VSMCs.^46^ To investigate the therapeutic potential of NAD^+^ boosting mitochondrial metabolism in familial aortic aneurysms, we treated *shFbn1* VSMCs with NR. Exposure of *shFbn1* VSMCs to NR for 5 days increased the expression of *Pparg1a* and *Tfam*, correlating with increased mtDNA content and the expression of the mtDNA-encoded *Mt-Co1* transcript (Figure 7A and 7B). NR increased OCR and decreased lactate production in *shFbn1* VSMCs to levels observed in *ShControl* VSMCs (Figure 7C and 7D). NR treatment of *shFbn1* VSMCs decreased the expression and activity of the proremodeling matrix metalloproteinases Mmp9 and Mmp2 and the profibrotic genes *Spp1* and *Col1a1* (collagen, type 1, α1; Figure 7E and 7F).

To confirm the potential of boosting mitochondrial metabolism to revert MFS features in humans, we performed in vitro analysis in primary skin fibroblast cultures from 4 healthy donors and 4 MFS patients. NR treatment restored the mRNA levels of *TFAM* and mtDNA (Figure 7G). NR increased the mRNA levels of *MT-CO1, MT-ND6*, and *HIF1A* (Figure 7G and 7H), restored OCR, and decreased lactate production in primary skin fibroblasts from MFS patients (Figure 7I and 7J). Furthermore, NR reduced the expression of the ECM genes COL1A1 and ACAN (aggrecan) and the profibrotic factors TGFB3 and CCN2 (connective tissue growth factor; Figure 7K). These data indicate that incubation with NR restores TFAM levels and improves mitochondrial respiration in vitro in cells from MFS patients.

### NR Treatment Reverts Aortic Aneurysm in a Mouse Model of MFS

**Figure 8.**
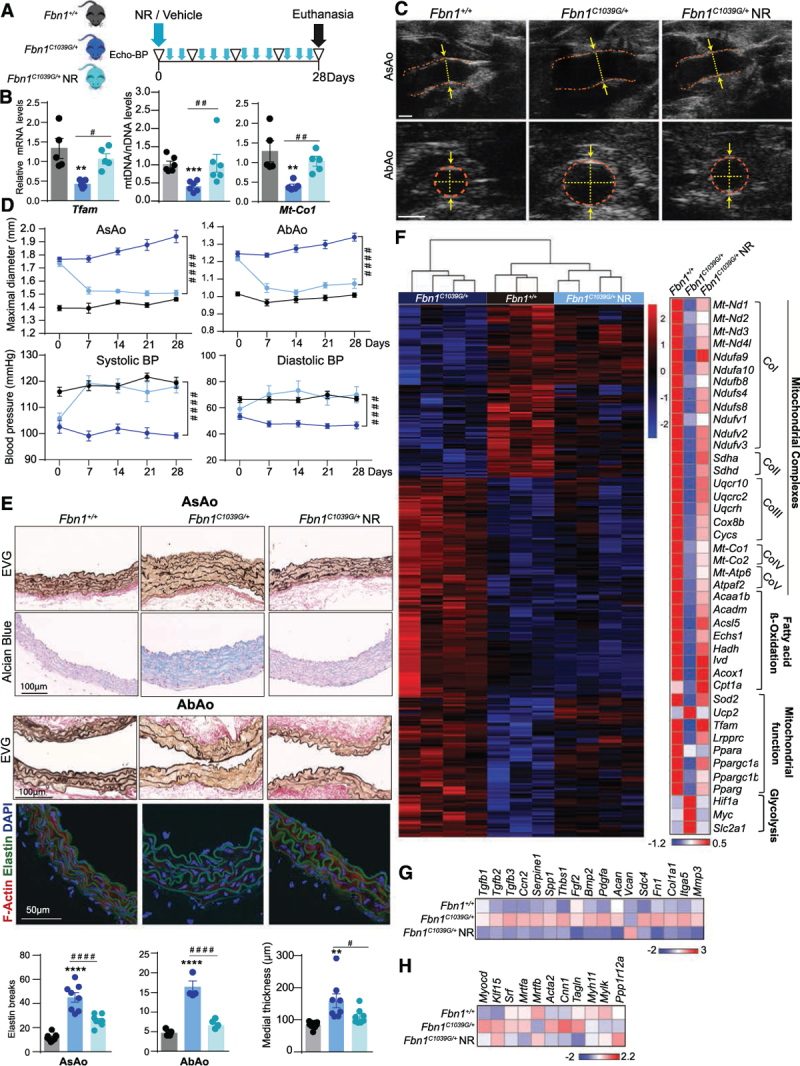
**Nicotinamide riboside treatment restores aortic homeostasis in a mouse model of Marfan syndrome.** **A**, Experimental design: 20-week-old *Fbn1*^+/+^ and *Fbn1*^*C1039G/+*^ male mice were treated with NR or vehicle for 28 days (blue arrowheads). Ultrasound and BP analysis was performed 5 times (empty triangles); n=9. **B**, Quantitative reverse transcription polymerase chain reaction analysis of *Tfam*, quantitative polymerase chain reaction analysis of relative mtDNA content, and *mt-Co1* mRNA. **C**, Representative aortic ultrasound images after 28 days of vehicle or NR treatment. Discontinuous red lines mark the lumen boundary, and discontinuous yellow lines and arrows denote the lumen diameter. **D**, Evolution of maximal AsAo and AbAo diameter (**top**) and systolic and diastolic BP (**bottom**) on NR treatment; n=9. **E**, Representative histologic staining with EVG and Alcian blue in the AsAo (**top**) and with EVG in the AbAo (**middle**) and confocal imaging of F-actin (red), elastin (green, autofluorescence), and 4′,6-diamidino-2-phenylindole–stained nuclei (blue) in the descending thoracic aorta (**bottom**); and quantification of elastin breaks and aortic medial thickness. **F** through **H**, RNA-sequencing analysis of aortic medial tissue from *Fbn1*^+/+^ mice (n=3) and from *Fbn1*^*C1039G/+*^ mice treated for 28 days with vehicle (n=4) or NR (n=4). **F**, Hierarchical clustering showing the top 200 most significant differentially expressed genes (by adjusted *P* < 0.05) between the 3 groups of mice (**left**) and gene expression heatmap for mitochondrial complex, fatty acid β-oxidation, mitochondrial function, and proglycolytic genes (**right**). **G**, Expression heatmap for genes encoding extracellular matrix–related proteins and (**H**) smooth muscle contractile apparatus. The heatmap was obtained from DESeq2 analysis. Statistical significance was assessed by 1-way ANOVA (**B**, **E**) or 2-way repeated measurements ANOVA (**D**). **P<0.01, ***P<0.001, ****P<0.0001 for *Fbn1*^*C1039G/+*^ vs *Fbn1*^+/+^; #*P*<0.05, ##*P*<0.01, ####*P*<0.0001 for *Fbn1*^*C1039G/+*^NR vs *Fbn1*^*C1039G/+*^. AbAo indicates abdominal aorta; Acaa1b, acetyl-Coenzyme A acyltransferase 1B; Acadm, acyl-CoA dehydrogenase medium chain; ACAN, aggrecan; Acsl5, Acyl-CoA Synthetase Long Chain Family Member 5; Acox1, acyl-Coenzyme A oxidase 1, palmitoyl; Acta2, actin, alpha 2, smooth muscle; AsAo, ascending aorta; Bmp 2, bone morphogenetic protein 2; BP, blood pressure; Ccn2, cellular communication network factor 2; Col1a1, collagen type I α1 chain; Cox8b, cytochrome c oxidase subunit 8B; Cnn1, calponin 1; Cpt1a, carnitine palmitoyltransferase 1A; Cycs, cytochrome c, somatic; DAPI, 4′,6-diamidino-2-phenylindole; Echs1, enoyl-CoA hydratase, short chain 1; EVG, elastin Van Gienson; F-Actin, filamentous actin; Fbn1, fibrillin-1; Fgf2, fibroblast growth factor 2; Fn1, Fibronectin1; Hadh, hydroxyacyl-CoA dehydrogenase; Hif1a, hypoxia-inducible factor 1 α; Itga5, integrin subunit alpha 5; Ivd, isovaleryl-CoA dehydrogenase; Klf15, Kruppel-like factor 15; Lrpprc, leucine rich pentatricopeptide repeat containing; Mmp3, matrix metalloproteinase 3; Mrtfa, b, myocardin related transcription factor A,B; Mt-Atp6, mitochondrially-encoded ATP synthase membrane subunit 6; Mt-Co1, 2, mitochondrially encoded cytochrome c oxidase I,II; Mt-Nd1–4l, mitochondrially-encoded NADH-ubiquinone oxidoreductase chains 1–4l, respectively; Myc, MYC proto-oncogene, bHLH transcription factor; Myh11, myosin heavy chain 11; Mylk, myosin light chain kinase; Myocd, myocardin; Ndufa9, 10, NADH:ubiquinone oxidoreductase subunit A9, 10; Ndufb8, NADH:ubiquinone oxidoreductase subunit B8; Ndufs4, 8, NADH:ubiquinone oxidoreductase core subunit S4; Ndufv1–3, NADH:ubiquinone oxidoreductase core subunit V1, V3; NR, nicotinamide riboside; Pdgfa, platelet derived growth factor subunit A; Pparg, Peroxisome proliferator-activated receptor gamma; Ppara, peroxisome proliferator-activated receptor α; Ppargc1a, b, peroxisome proliferator activated receptor γ coactivators 1a and b, respectively; Ppp1r12a, protein phosphatase 1 regulatory subunit 12A; Sdc4, Syndecan4; Sdha, d, succinate dehydrogenase complexes A and D, respectively; Serpine1, serpin family E member 1; Slc2a1, solute carrier family 2 member 1; Spp1, secreted phosphoprotein 1; Sod2, superoxide dismutase 2, mitochondrial; Srf, serum response factor; Tagln, transgelin; Tfam, mitochondrial transcription factor A; Tgfb1–3, transforming growth factors β1–3, respectively; Thbs1, thrombospondin 1; Uqcr10, ubiquinol-cytochrome c reductase, complex III subunit X; Uqcrc2, ubiquinol-cytochrome c reductase core protein 2; Uqcrh, ubiquinol-cytochrome c reductase hinge protein; and Vcan, Versican.

We next tested the therapeutic potential of NR to prevent or even revert the development of aneurysm in MFS mice by modulating mitochondrial metabolism. Starting at 20 weeks old, *Fbn1*^*C1039G/+*^ mice received IP injections of NR every second day for 28 days (Figure 8A; Figure VA in the Data Supplement). NR restored aortic *Tfam* and *Mt-Co1* mRNA expression and mtDNA content to normal levels in MFS mice (Figure 8B). Importantly, aortic dilation and BP were completely normalized after 7 days of treatment in both male and female mice (Figure 8C and 8D; Figure VB in the Data Supplement). Moreover, NR restored histologic features of aortic degeneration in MFS, such as medial thickening, elastic fiber fragmentation, proteoglycan deposition, and actin polymerization (Figure 8E; Figure VC in the Data Supplement).

To characterize the effect of NR treatment at the molecular level, we performed RNA-sequencing on aortas from MFS and control mice. Hierarchical clustering of the transcriptomic analysis revealed that NR reverted the transcriptional changes observed in *Fbn1*^*C1039G/+*^ aortas, bringing expression levels closer to controls than to *Fbn1*^*C1039G/+*^ mice (Figure 8F). NR increased the expression levels of genes related to mitochondrial complexes, fatty acid β-oxidation, and mitochondrial function, including *Tfam* and *Ppargc1a*, and reduced the expression of genes related to glycolytic rewiring, such as *Hif1a* and *Myc* (Figure 8F). Notably, NR also decreased the expression of classical MFS-affected genes such as transcripts involved in the TGFβ pathway and those encoding ECM-related proteins (Figure 8G). Additionally, NR brought the expression of some transcription factors involved in the maintenance of the contractile phenotype and genes related to the smooth muscle contraction apparatus, such as smooth muscle *Acta2* and *Cnn1*, down to *Fbn1*^+/+^ (Figure 8H). Thus, boosting mitochondrial function with NR rapidly restores the transcriptional signature in aortas from MFS mice and fully reverts the associated aortic wall remodeling, aortic dilation, and medial degeneration. Taken together, our data highlight VSMC metabolism as a critical mediator of hereditable TAAs and suggest that the use of mitochondrial-boosting compounds is a promising pharmacologic strategy for treating patients with these hereditary disorders.

## Discussion

We found that aortas from *Fbn1*^*C1039G/+*^ mice and human MFS patients present low TFAM expression, below-normal mtDNA levels, and a decline in mitochondrial respiration. This decline in oxidative phosphorylation was compensated by increased glycolytic metabolism and appears at the onset of the aortic disease. In line with our data, aortas from the *Cutis laxa* mouse model showed decreased mitochondrial respiration and increased glycolysis,^47^ suggesting that mitochondrial decline could be a common driver and hallmark of different hereditary TAAs.

Next, we tried to elucidate how aneurysms caused by mutations related to ECM and VSMC contractile function converge on the impairment of TFAM expression and mitochondrial dysfunction. Recent reports in epithelial cells show that stiff matrices produce a mechanical regulation of glycolysis via remodeling of the cytoskeleton architecture.^22^ Because VSMCs and aortas carrying TAA mutations show an increase of ECM deposition, active peptides and growth factors, stiffness, and mechanotransduction,^17,18,48^ we hypothesized that the ECM could drive this metabolic rewiring toward glycolysis. In vitro experiments in VSMCs seeded in matrices produced by Fbn1-deficient VSMCs support that metabolism is controlled by the ECM. Hence, changes in the composition of the ECM during aneurysm development drives metabolic rewiring toward glycolysis. In epithelial cells, PI3K (phosphoinositide 3-kinase) signaling coordinates glycolytic metabolism and actin remodeling. Activation of PI3K mobilizes aldolase from F-actin, increasing glycolysis.^20,49^ In endothelial cells, another glycolytic pathway enzyme, PFKFB (phospho-fructokinase 2/fructose-2,6 bisphosphatase), regulates vessel sprouting through the coordination of glycolytic metabolism and the actin cytoskeleton.^50^ In dermal fibroblasts, downregulation of fatty acid β-oxidation and an increased glycolysis rate contribute to ECM alterations, promoting fibrosis.^51^ In VSMCs from pulmonary hypertension patients, inhibiting glycolysis by silencing α-enolase prevents the malignant secretory phenotype.^52^ Altogether, these data support that there is a bidirectional connection between the ECM composition and the cellular metabolism with a critical implication in aortic tissue homeostasis (Figure VI in the Data Supplement)

To demonstrate the importance of VSMC metabolism in the development of aneurysms, we generated a conditional mouse model with specific deletion of Tfam in VSMCs. Tfam deficiency in VSMC promotes mitochondrial dysfunction and a metabolic rewiring toward glycolysis. Tfam-depleted VSMCs acquire a senescent and proinflammatory phenotype together with an impairment in their contractile function. Moreover, these mice develop aortic aneurysm, medial degeneration, and lethal dissections. Similarly, silencing of Tfam in fibroblast prevents differentiation toward myofibroblasts and the acquisition of a contractile phenotype.^53^ During the last decade, it became evident that signals from stressed mitochondria regulate senescence and inflammatory cascades in multiple cell types.^36,37,54^ The release of mtDNA from the mitochondria to the cytosol activates the cGAS-STING response and contributes to the progression of the aneurysm.^37,38^ In the same line, we have observed the activation of cGAS-STING route in both in aortas from *Fbn1*^*C1039G/+*^ mice and in *SM-Tfam*^−/−^ mice, further confirming the involvement of this route in the pathogenesis of the aortic diseases.

Besides the high incidence and mortality risk of ruptured aneurysms in inherited TAA patients, there are still limited therapeutic options to delay TAA progression to dissection and none to prevent it. Current pharmacologic standard treatments for TAA are based on BP control with β-adrenergic blockers or the Ang II receptor-1 antagonist losartan, which slow down aortic dilation but do not prevent dissection.^55,56^ Therefore, the current management of aortic aneurysms relies on surgical prophylactic repair, which shows significantly higher perioperative mortality and morbidity.^57^ Consequently, it is imperative to identify novel molecular mediators involved in hereditable TAA pathophysiology to design new pharmacologic strategies. Since the use of NAD^+^ precursors has emerged as an effective approach to boost mitochondrial dysfunction in different pathologies,^40,41,45^ we investigated the pharmacologic potential of NR in TAAs associated with genetic disorders. We observed that NR rapidly raised Tfam levels, improved mitochondrial metabolism, and normalized aortic function and diameter in the Marfan mouse model of TAA. Our results indicate that glycolytic metabolism is a common driver of hereditary TAAs and NR could be trialed for the treatment of different types of genetic disorders that cause TAA. Further research would be required to determine if this approach could also be effective against other vasculopathies, such as cerebral aneurysm or pulmonary hypertension.

## Acknowledgments

We thank N.G. Larsson for *Tfam*^*fl/fl*^ mice, Dr S. Offermans (Max Planck Institute for Heart and Lung Research, Bad Nauheim, Germany) for supplying mice expressing tamoxifen-inducible Cre recombinase specifically in VSMCs (*Myh11-Cre*^*ERT2*^), and S. Bartlett for English editing. We also thank the CNIC imaging facility, A.V. Alonso, and L. Flores for technical support. The genomic data analysis and statistics were performed by the Genomics and NGS Core Facility at the Centro de Biología Molecular Severo Ochoa (CBMSO, CSIC-UAM) which is part of the CEI UAM+CSIC, Madrid, Spain.

J.O. and M.M. designed the research. J.O. performed most of the experiments and analyzed the data. R.R-D., C.B-M. and A.M.B. performed contractility assays. E.G-R., G.D.M, J.F.A. R.R.M., P.A., E.M.B., and M.J.R-R. provided experimental and technical support. A.F.G., C.E.M.L., J.L.M-V., J.F.N., J.M.R., C.L.L.C., and M.E.L. provided reagents. J.O., E.G-R., and M.M. wrote the manuscript.

## Sources of Funding

This study was supported by the Fondo de Investigación Sanitaria del Instituto de Salud Carlos III (PI16/188, PI19/855), the European Regional Development Fund, and the European Commission through H2020-EU.1.1, European Research Council grant ERC-2016-StG 715322-EndoMitTalk, and Gobierno de España SAF2016-80305P. This work was partially supported by Comunidad de Madrid (S2017/BMD 3867 RENIM-CM) and cofinanced by the European Structural and Investment Fund. M.M. is supported by the Miguel Servet Program (CP 19/014, Fundación de Investigación del Hospital 12 de Octubre). J.O., E.G., and R.R-D. are supported by Juan de la Cierva (FJCI2017-33855, IJC2018-036850-I, and IJCI-2017-31399, respectively). Support was also provided by Ministerio de Ciencia e Innovación grants (RTI2018-099246-B-I00 to J.M.R. and PI18/00543 to J.F.N.) and Comunidad de Madrid and Fondo Social Europeo funds (AORTASANA-CM; B2017/BMD-3676 to A.M.B., A.F., and J.M.R.). J.M.R. was also funded by Fundacion La Caixa (HR18-00068) and the Marfan Foundation (USA). J.M.R. and J.L.M.V. were also funded by Centro de Investigación Biomedica en Red Enfermedades Cardiovasculares of Ministerio de Ciencia e Innovación (CB16/11/00264). J.F.N. was funded by Ministerio de Economía y Competitividad (PI18/00543) and Centro de Investigación Biomedica en Red Enfermedades Cardiovasculares (CB16/11/00264), and was cofunded by Fondo Europeo de Desarrollo Regional.

## Disclosures

None.

## Supplemental Materials

Data Supplement Figures I–VI

Data Supplement Table I

## Supplementary Material


