# Supplementary material for: Extracellular Tuning of Mitochondrial Respiration Leads to Aortic Aneurysm
Source: Circulation. 2021 Mar 12;143(21):2091–109. doi: 10.1161/CIRCULATIONAHA.120.051171 (PMC8140666; doi:10.1161/CIRCULATIONAHA.120.051171)
Supplement: Supplementary file 1 [file cir-143-2091-s001.pdf]

SUPPLEMENTAL MATERIAL

Expanded Methods

Supplemental Table I

Real-time quantitative qPCR was performed with the following primers:

Mouse Primers		
**Gene**	**Forward**	**Reverse**
Acta2	ATCGTCCACCGCAAATGC	AAGGAACTGGAGGCGCTG
Col1a1	GCTCCTCTTAGGGGCCACT	CCACGTCTCACCATTGGGG
Cnn1	AACTTCATGGATGGCCTCAAA	ACCCGGCTGCAGCTTGT
Fbn1	GGAATGACATCAGCAGGCAC	TACACAAATCCTTTGGGGCA
Hif1a	AGCTTCTGTTATGAGGTCAGC	TGACTTGATGTTCATCGTCC
Mmp2	CAAGTTCCCCGGCGATGTC	TTCTGGTCAAGGTCACCTGTC
Mmp9	CACCACAGCCAACTATGACCA	CAGGAAGACGAAGGGGAAGAC
Myh11	TCAACGCCAACCGCAGGAAGCTG	TGCTAAGCAGTCTGCTGGGCT
mt-Co1	CTCGCCTAATTTATTCCACTTCA	GGGGCTAGGGGTAGGGTTAT
mt-Nd1	CTAGCAGAAACAAACCGGGC	CCGGCTGCGTATTCTACGTT
Nos2	CTTTGCCACGGACGAGAC	TCATTGTACTCTGAGGGCTGAC
Smtn	TCACTACCTTCAGCCATGCCT	GCCATTAGCTGCTTCCACTGT
Spp1	ATGAGATTGGCAGTGATTTG	CATCCTTTTCTTCAGAGGAC
Tagln	CTATGAAGGTAAGGATATGGC	TCTGTGAAGTCCCTCTTATG
Tfam	CAGGAGGCAAAGGATGATTC	CCAAGACTTCATTTCATTGTCG
Ppargc1a	GGCACGCAGCCCTATTCA	CGACACGGAGAGTTAAAGGAAGA
**At least two different reference targets among these were used for normalization:**		
b2m	TACATACGCCTGCAGAGTTAAGCA	TGATCACATGTCTCGATCCCAG)
pp1a	ACGCCACTGTCGCTTTTC	GCAAACAGCTCGAAGGAGAC
Ywhaz	TTACTTGGCCGAGGTTGCT	TGCTGTGACTGGTCCACAAT
Bactin	CTAAGGCCAACCGTGAAAAG	ACCAGAGGCATACAGGGACA
**for mtDNA**		
mt-Co1	CTCGCCTAATTTATTCCACTTCA	GGGGCTAGGGGTAGGGTTAT
mt-Nd1	CTAGCAGAAACAAACCGGGC	CCGGCTGCGTATTCTACGTT
16s Mt-rRNA	CCGCAAGGGAAAGATGAAAGAC	TCGTTTGGTTTCGGGGTTTC
**For nDNA**		
Hk2	GCCAGCCTCTCCTGATTTTAGTGT	GGGAACACAAAAGACCTCTTCTGG

Human primers		
**Gene**	**Forward**	**Reverse**
ACAN	TCGAGGACAGCGAGGCC	TCGAGGGTGTAGCGTGTAGAGA
CCN2	CGACTGGAAGACACGTTTGG	AGGCTTGGAGATTTTGGGAG
COL1A1	ATGTCTAGGGTCTAGACATGTTCA	CCTTGCCGTTGTCGCAGACG
CYCS	-GCCTGCCTGATCCTCCAAAT	AAGGTAGCGGATGATTCAGCC-
HIF1A	GTGGATTACCACAGCTGA	GCTCAGTTAACTTGATCCA
MMP2	CTCAGATCCGTGGTGAGATCT	CTTTGGTTCTCCAGCTTCAGG

MMP9	ATCCAGTTTGGTGTCGCGGAGC	GAAGGGGAAGACGCACAGCT
MYC	AAACACAAACTTGAACAGCTAC	ATTTGAGGCAGTTTACATTATGG
MT-ATP6	TAGCCATACACAACACTAAAGGACGA	GGGCATTTTTAATCTTAGAGCGAAA
MT-COI	GACGTAGACACACGAGCATATTTCA	AGGACATAGTGGAAGTGAGCTACAAC
MT-ND1	CCACCTCTAGCCTAGCCGTTTA	GGGTCATGATGGCAGGAGTAAT
PPARA	CTATCATTTGCTGTGGAGATCG	AAGATATCGTCCGGGTGGTT
PPARG	GAGAAGGAGAAGCTGTTGGC	ATGGCCACCTCTTTGCTCT
PPARGC1A	GGCAGAAGGCAATTGAAGAG	TCAAAACGGTCCCTCAGTTC
SDHA	TGGGAACAAGAGGGCATCTG	CCACCACTGCATCAAATTCATG
SDHB	GACACCAACCTCAATAAG	GATTCATCCTTCTTCTTCAA
TGFB3	TGAGTGGCTGTTGAGAAGAGA	ATTGTCCACGCCTTTGAATTTGAT
TFAM	CCGAGGTGGTTTTCATCTGT	GCATCTGGGTTCTGAGCTTT
**At least two different reference targets among these were used for normalization:**		
UBC	ATTTGGGTCGCGGTTCTTG	TGCCTTGACATTCTCGATGG
GADPH	GAAGGTGAAGGTCGGAGTC	GAAGATGGTGATGGGATTTC
BACTIN	GATCATTGCTCCTCCTGAGC	ACATCTGCTGGAAGGTGGAC
**mtDNA**		
CYCS	GCCTGCCTGATCCTCCAAAT	AAGGTAGCGGATGATTCAGCC-
MT-COI	CCACCTCTAGCCTAGCCGTTTA	GGGTCATGATGGCAGGAGTAAT
16s MT-LEU-tRNA	CACCCAAGAACAGGGTTTGT	TGGCCATGGGTATGTTGTTA
**nDNA**		
B2M	TGCTGTCTCCATGTTTGATGTATCT	TCTCTGCTCCCCACCTCTAAGT

Supplemental Figures and Figure Legends

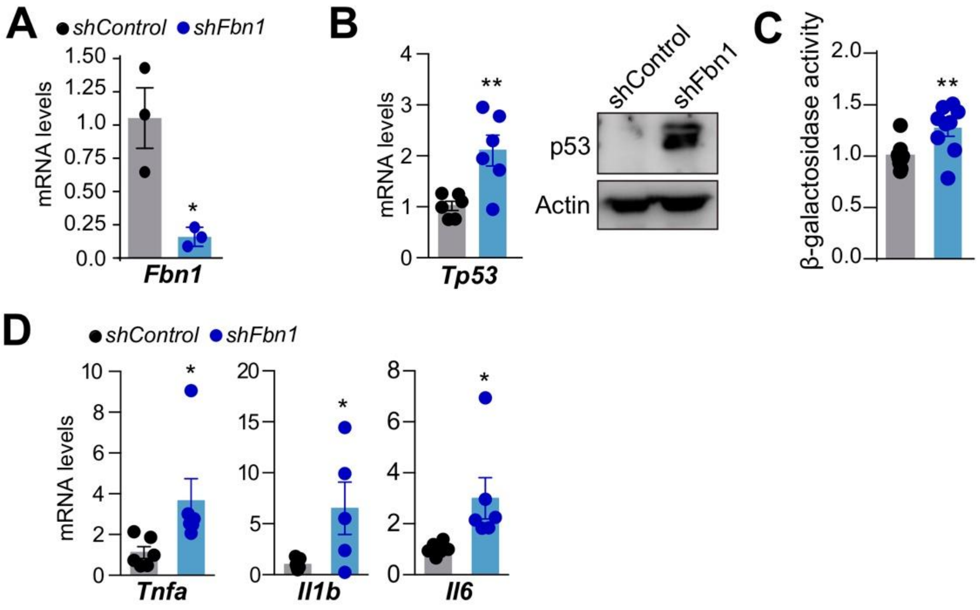

Supplemental Figure I: Senescent and inflammatory phenotype in *Fbn1*-deficient VSMCs.

Primary VSMCs were transduced with *shFbn1* as *in vitro* model of MFS. (**A**) RT-qPCR analysis of *Fbn1*. (**B**) RT-qPCR (n=6) and representative inmunoblot (out of 4) analysis of *p53* (n=4). (**C**) Relative senescent associated B-galactosidase activity. (**D**) RT-qPCR analysis of pro-inflammatory genes *Tnfa*, *Il1b* and *Il6*. Data are mean ± s.e.m. Statistical significance was assessed by Student's t-test *P < 0.05, **P < 0.01 vs *shControl*.

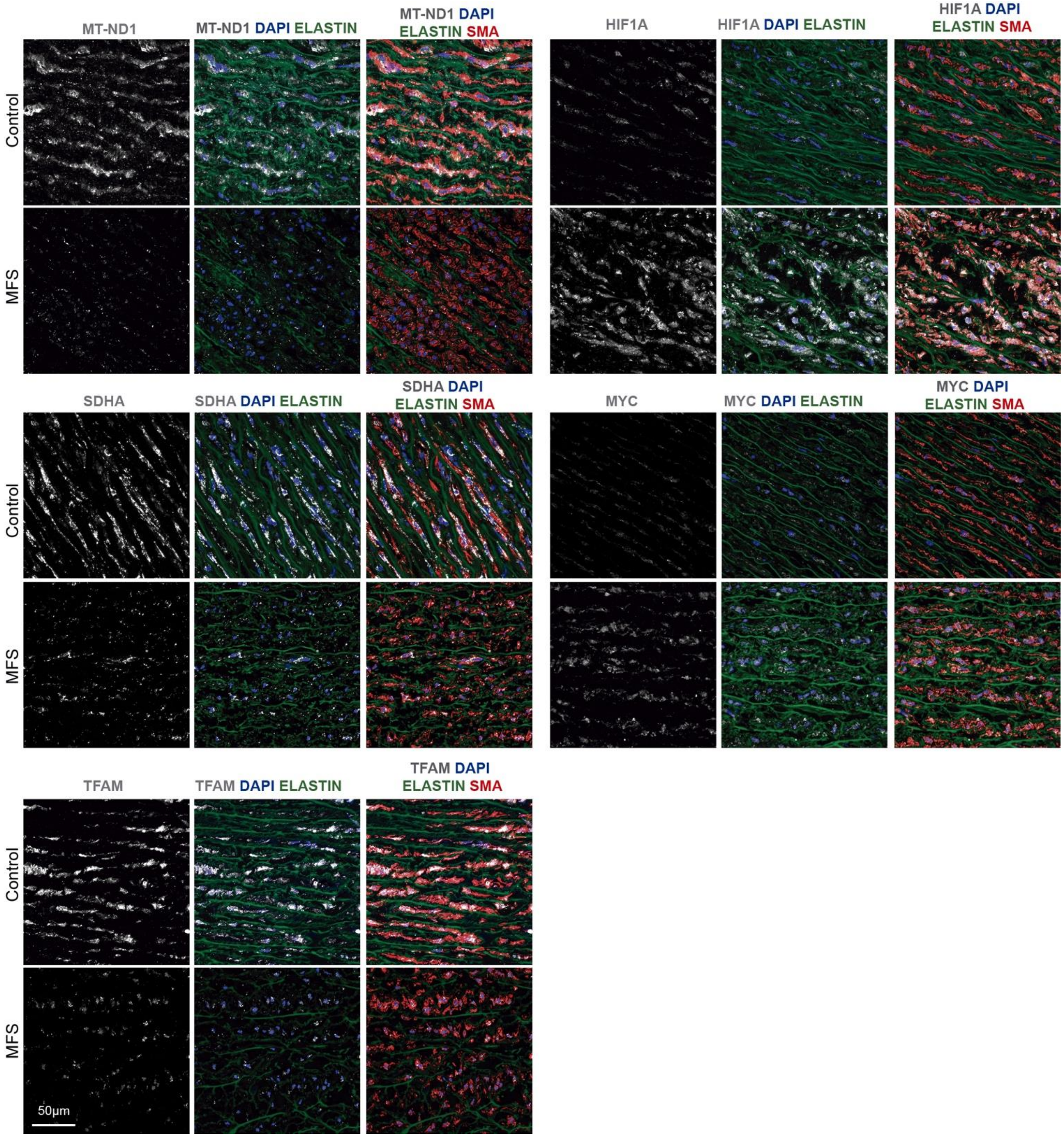

Supplemental Figure II: Immunofluorescence analysis of metabolic proteins in human aortic media from MFS patients.

Representative medial layer sections from ascending aortas of control donors and MFS patients, showing immunofluorescence confocal analysis for MT-CO1 (CoIV), SDHA (CoII), TFAM HIF1A, and MYC (white); SMA (red), and elastin (green autofluorescence).

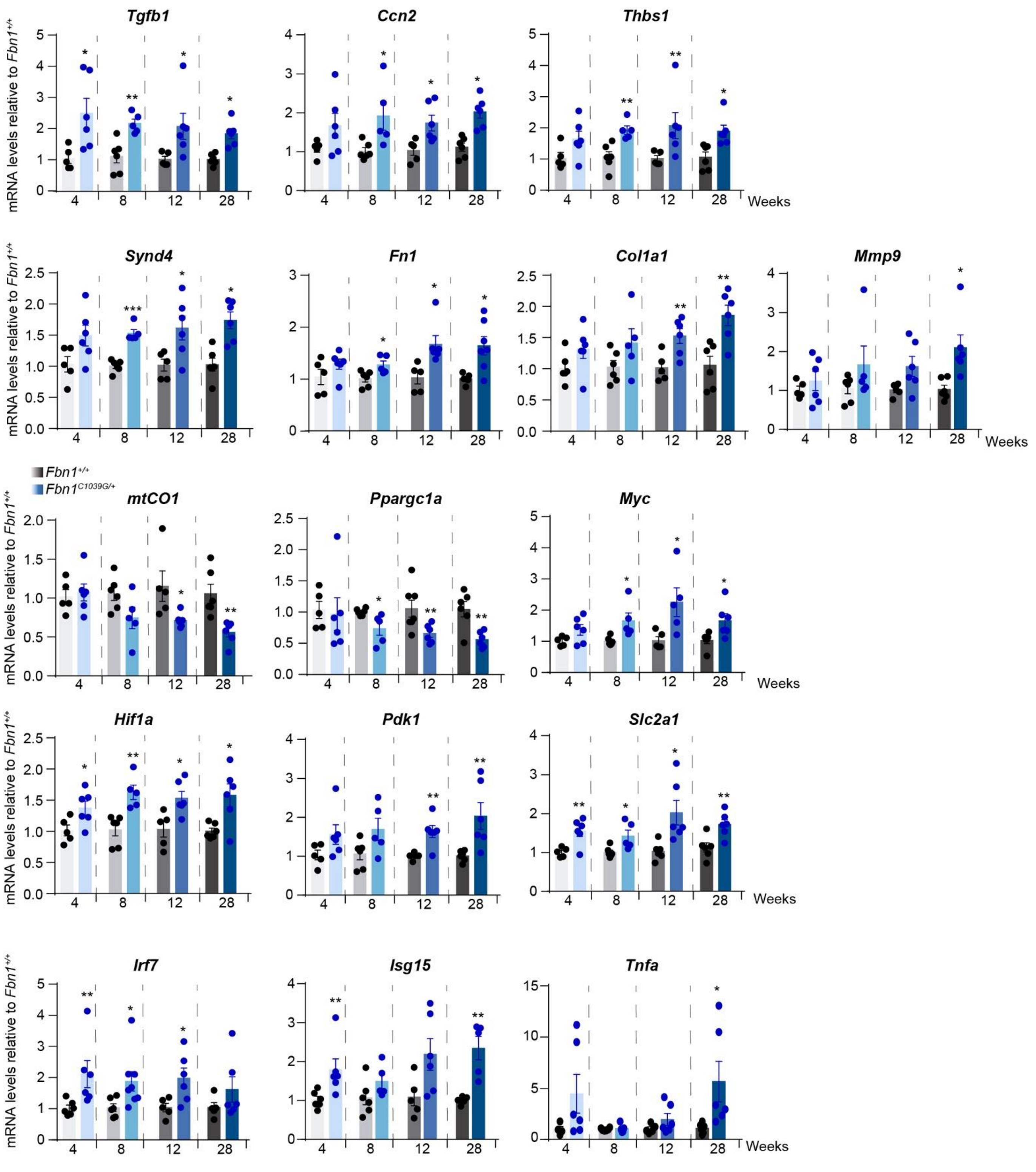

Supplemental Figure III: Gene expression profile at different ages in *Fbn1*$^{C1039G/+}$ mice

RT-qPCR analysis of indicated genes in aortas from *Fbn1*$^{+/+}$ and *Fbn1*$^{C1039G/+}$ mice at different ages. Statistical significance was assessed by Student's t-test *P < 0.05, **P < 0.01, ***P < 0.001 vs *Fbn1*$^{+/+}$.

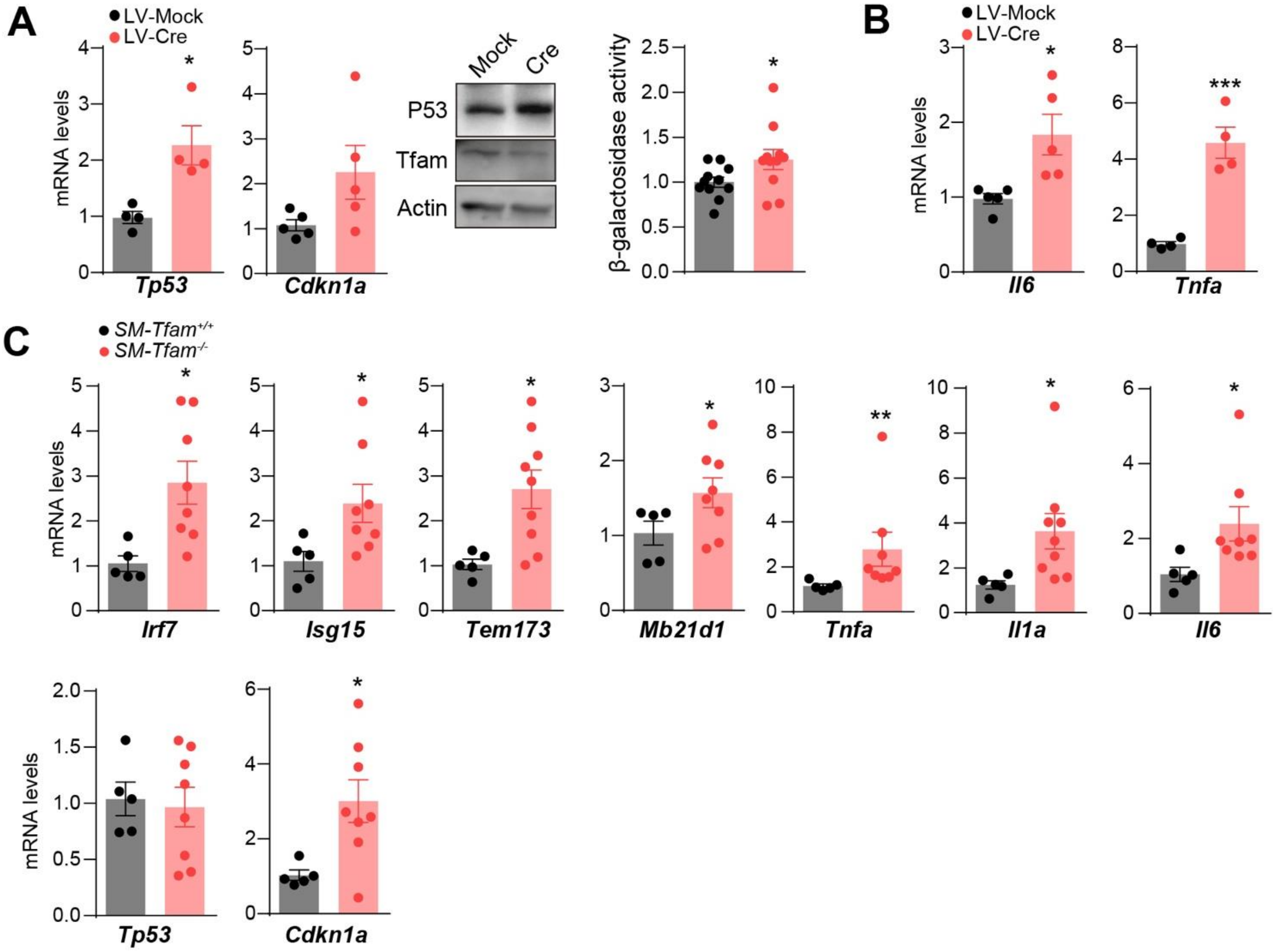

Supplemental Figure IV: Senescent and inflammatory phenotype in *Tfam*-deficient VSMCs.

(A-B) Primary mouse *Tfam*$^{flox/flox}$ VSMCs were transduced with GFP-expressing (LV-Mock) or Cre-expressing (LV-Cre) lentivectors and analyzed after fourteen days. (**A**) RT–qPCR analysis of relative *Tp53,* and *Cknd1a* and representative inmunoblot of P53. Actin was used as total protein loading control. (**B**) RT–qPCR analysis of relative *Il6,* and *Tna.* (C) *SM-Tfam*$^{+/+}$ and *SM-Tfam*$^{-/-}$ mice were treated with Tmx at five weeks of age. **(C)** RT-qPCR analysis of the indicated genes in aortic extracts twelve weeks after Tmx. Statistical significance was assessed by Student's t-test *P < 0.05, **P < 0.01, ***P < 0.001 vs Lv-Mock (A,B) or *SM-Tfam*$^{+/+}$.

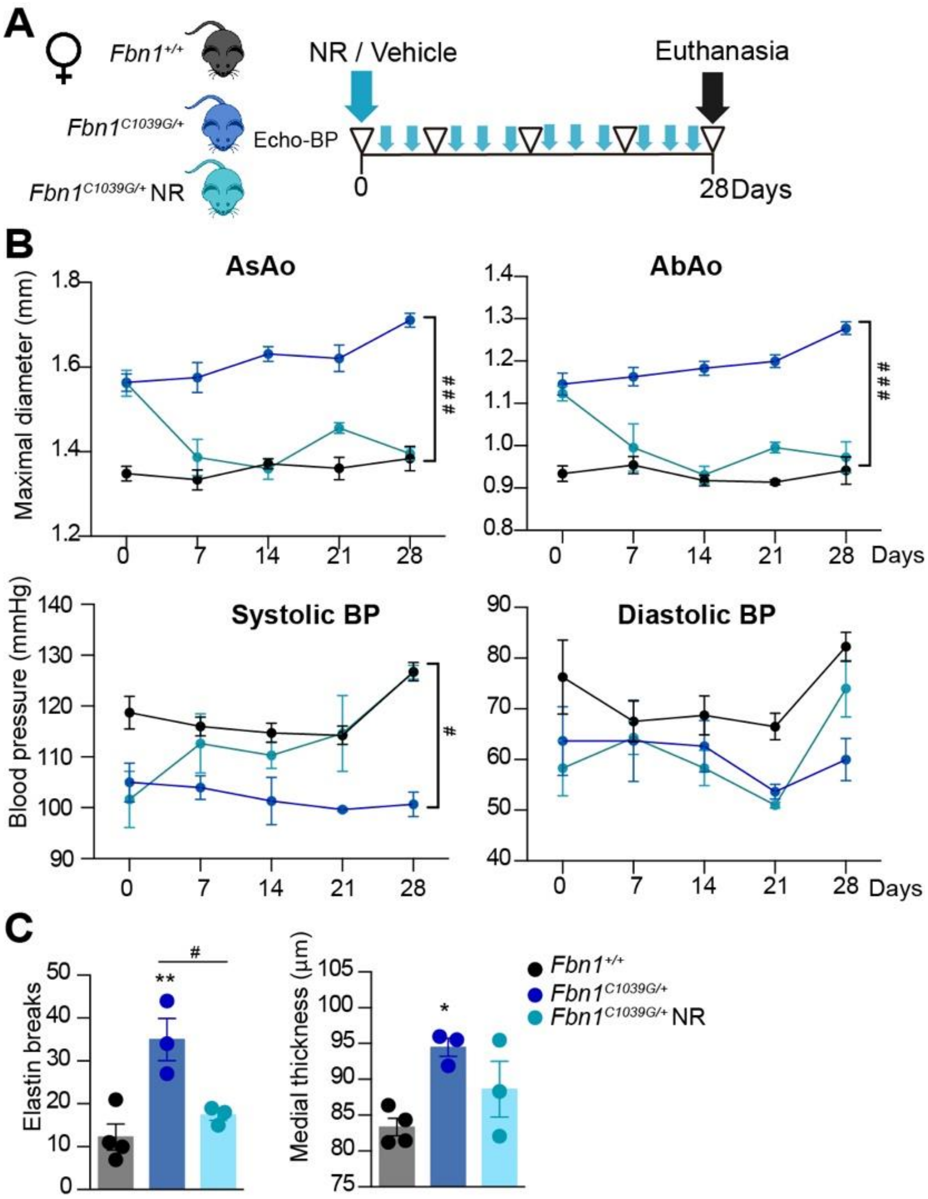

Supplemental Figure V: NR treatment reverts aortic dilation in female MFS mice

(**A**) Experimental design: 16-week-old *Fbn1*$^{+/+}$ and *Fbn1*$^{C1039G/+}$ female mice were treated with vehicle or NR for 28 days as follows: n=4 *Fbn1*$^{+/+}$ mice; n=3 vehicle-treated *Fbn1*$^{C1039G/+}$ mice; n=3 NR-treated *Fbn1*$^{C1039G/+}$ mice. Ultrasound and BP analysis was performed five times (empty triangles). (**B**) Maximal AsAo and AbAo diameters and systolic and diastolic BP after NR treatment. (**C**) Quantification of AsAo elastin breaks and aortic medial thickness. Statistical significance was assessed by two-way repeated measurements ANOVA (B) or one-way ANOVA (C). *P < 0.05, **P < 0.01, vs *Fbn1*$^{+/+}$; # P < 0.05, ###P < 0.001, for *Fbn1*$^{C1039G/+}$ vs *Fbn1*$^{C1039G/+}$ NR.

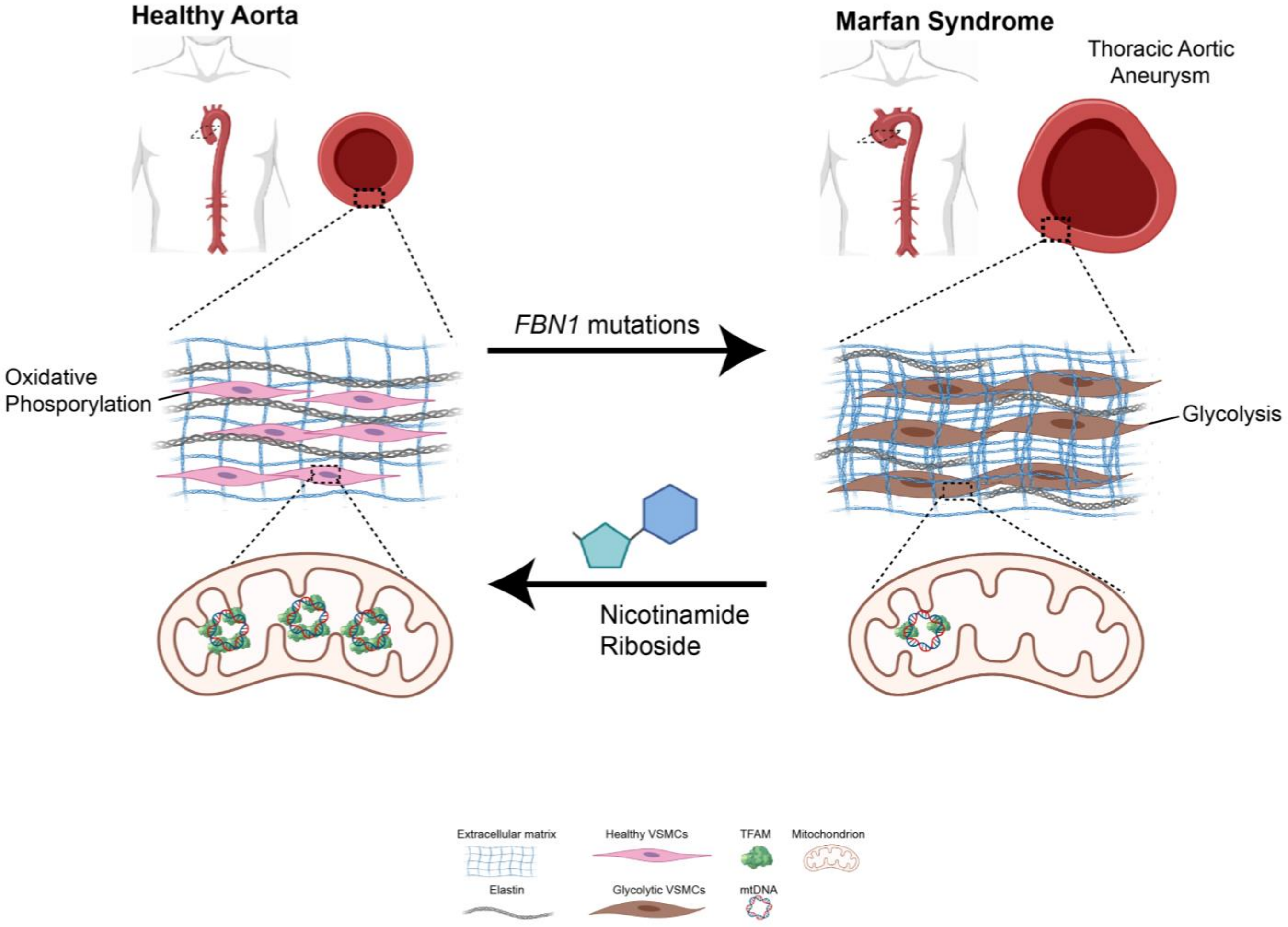

Supplemental Figure VI: Proposed model depicting the critical role of mitochondrial decline in promoting aortic alterations in Marfan syndrome.

Marfan syndrome (MFS) is an autosomal dominant disorder caused by mutations in the extracellular protein FBN1. The major complication of MFS is the development of thoracic aortic aneurysms, which are characterized by aortic dilation and extracellular matrix (ECM) remodeling. ECM from MFS cells modulates VSMC metabolism promoting a switch from mitochondrial respiration to glycolysis. Glycolytic VSMCs acquire a senescent and inflammatory phenotype with decreased contractile capacity favoring the development of the aneurysm. Restoring mitochondrial metabolism with the NAD-precursor nicotinamide riboside rapidly reverts aortic aneurysm in *Fbn1*$^{c1039g/+}$ mice.
